# Distributed feedforward and feedback cortical processing supports human speech production

**DOI:** 10.1073/pnas.2300255120

**Published:** 2023-10-11

**Authors:** Ran Wang, Xupeng Chen, Amirhossein Khalilian-Gourtani, Leyao Yu, Patricia Dugan, Daniel Friedman, Werner Doyle, Orrin Devinsky, Yao Wang, Adeen Flinker

**Affiliations:** ^a^Electrical and Computer Engineering Department, New York University, New York, NY 11201; ^b^Neurology Department, New York University, New York, NY 10016; ^c^Biomedical Engineering Department, New York University, New York, NY 11201; ^d^Neurosurgery Department, New York University, New York, NY 10016

**Keywords:** speech production, auditory feedback, brain computer interface, electrocorticography, speech motor control

## Abstract

Speech production is a complex human function requiring continuous feedforward commands together with reafferent feedback processing. These processes are carried out by distinct frontal and temporal cortical networks, but the degree and timing of their recruitment and dynamics remain poorly understood. We present a deep learning architecture that translates neural signals recorded directly from the cortex to an interpretable representational space that can reconstruct speech. We leverage learned decoding networks to disentangle feedforward vs. feedback processing. Unlike prevailing models, we find a mixed cortical architecture in which frontal and temporal networks each process both feedforward and feedback information in tandem. We elucidate the timing of feedforward and feedback–related processing by quantifying the derived receptive fields. Our approach provides evidence for a surprisingly mixed cortical architecture of speech circuitry together with decoding advances that have important implications for neural prosthetics.

## Significance

Human speech production is a complex behavior that involves feedforward control of motor commands as well as feedback processing of self-produced speech. These processes require the engagement of multiple brain networks in tandem. However, it has been hard to dissociate the degree and timing of cortical recruitment for motor control versus sensory processing generated by speech production. We introduce a neural network framework that reconstructs produced speech from neural activity and leverages the learned decoding networks to disentangle these processes. Unlike prevailing models, we find a surprisingly mixed feedforward and feedback cortical recruitment during speech production. These results, together with our speech decoding advances, have important implications for speech motor control and neural prosthetics.

The central sulcus divides the human frontal from the posterior temporal, parietal, and occipital neocortices (1). Traditionally, this divide separates high-order planning and motor execution from sensation. Feedforward execution lies in the frontal cortices in contrast to feedback sensory processing across association cortices for the various modalities (e.g., auditory, visual, somatosensory, etc.) (2). Higher-order capacities such as working memory, cognitive control, and decision-making are often viewed as initiated by frontal cortices with direct influence on sensory cortices (3–5).

Human higher-order cognitive functions include planning and executing complex speech sequences that carry semantic and linguistic meaning (6, 7). Speech production is a complex human motor behavior requiring precise coordination of multiple oral, laryngeal, and respiratory muscles (8). These finely tuned motor actions then produce reafferent feedback in the auditory, tactile, and proprioceptive domains as we process our own speech.

Prevailing models in human speech motor control propose a feedforward system that predicts and generates actions and a feedback system responding to the vocal auditory and somatosensory effects (9–14). Across these models, there is a consensus that the two systems are anatomically separated, with the feedforward system mainly supported by ventral frontal cortices, while feedback processing is carried out across various sensory cortices (e.g., Heschl’s and superior temporal gyri for auditory feedback). During speech production, both these systems are engaged in producing articulatory motor movements as well as perceiving the auditory (and somatosensory) feedback produced by our actions. A major challenge in human neuroimaging of speech circuitry is dissociating neural signatures that are due to the feedforward motor plan in contrast to feedback from auditory processing as well as elucidating the exact dynamics of feedforward and feedback engagement across peri-Sylvian cortex.

A growing literature has leveraged unique human electrocorticographic (ECoG) recordings from patients undergoing neurosurgical procedures to obtain a combined spatial and temporal resolution critical for investigating speech production. Studies have detailed the signatures of feedforward speech planning (15) and organization of execution (16–18) in frontal cortices as well as the subsequent auditory feedback architecture in temporal cortices (18–21). Recently, deep neural network approaches have been developed to decode speech represented in auditory (22–25) and sensorimotor (26, 27) cortices from ECoG recordings. Nevertheless, these approaches have not been able to disentangle feedforward and feedback contributions during speech production as the motor and sensory responses co-occur.

**Fig. 1.**
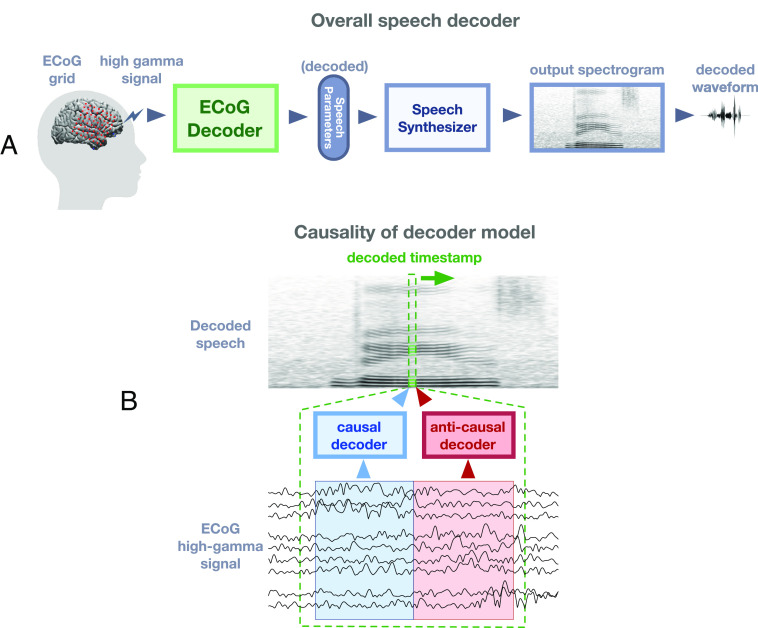
Speech decoding framework. (*A*) The overall structure of the decoding pipeline. ECoG amplitude signals are extracted in the high gamma range (i.e., 70–150 Hz). The ECoG Decoder translates neural signals from the electrode array to a set of speech parameters. This parameter representation is used to drive a speech synthesizer which creates a spectrogram (and associated waveform). (*B*) Illustration of causal versus anticausal neural decoding, which is leveraged to disentangle feedforward and feedback contributions. In order to reconstruct a speech timestamp, the causal model only uses feedforward neural signals in the past (blue), whereas the anticausal model only uses the neural signals that occur after the timestamp (red). The noncausal model is allowed to use both past and future neural signals to decode the current timestamp.

We directly disentangle feedback and feedforward processing during speech production by applying a deep learning architecture on human neurosurgical recordings to decode speech (Fig. 1*A*; *Materials and Methods, Visualizing Spatial Contribution Map*). Our approach decodes interpretable speech parameters from cortical signals, which drives a rule-based differentiable speech synthesizer. By learning neural network architectures which apply causal (predicting using only the past), anticausal (predicting using the future feedback), or both (noncausal), temporal convolutions (Fig. 1*B*), we are able to analyze the overall feedforward and feedback contributions, respectively, as well as to elucidate the temporal receptive fields of recruited cortical regions. This approach allows us to operationalize feedforward contributions related to the motor plan with our causal models. The feedback contributions related to hearing auditory feedback are operationalized with our anticausal models. In contrast to current views that separate feedback and feedforward cortical networks, our analyses reveal a surprisingly mixed architecture of feedback and feedforward processing both in frontal and temporal cortices while achieving superb speech decoding performance.

## Results

A major challenge in speech production research is dissociating neural signatures that are feedforward, or motor in nature, rather than auditory and somatosensory feedback produced by speech articulation. Given that these neural signals co-occur, we chose to disentangle their contributions by leveraging deep neural network architectures (i.e., Speech Decoding, Fig. 1*A*) that can decode speech acoustics from the neural responses in different temporal directions (i.e., feedforward and feedback, Fig. 1*B*; *Materials and Methods, Speech Decoding Framework*). We leverage neurosurgical ECoG data obtained from five participants who took part in a battery of tasks: Auditory Repetition (AR), Auditory Naming (AN), Sentence Completion (SC), Word Reading (WR), and Picture Naming (PN). These were designed to elicit the same set of spoken words across tasks while varying the stimulus modality (28) and provided 50 repeated unique words (400–800 total trials per participant), all of which were analyzed locked to the onset of speech production.

### Robust Speech Decoding from Neural Signals.

**Fig. 2.**
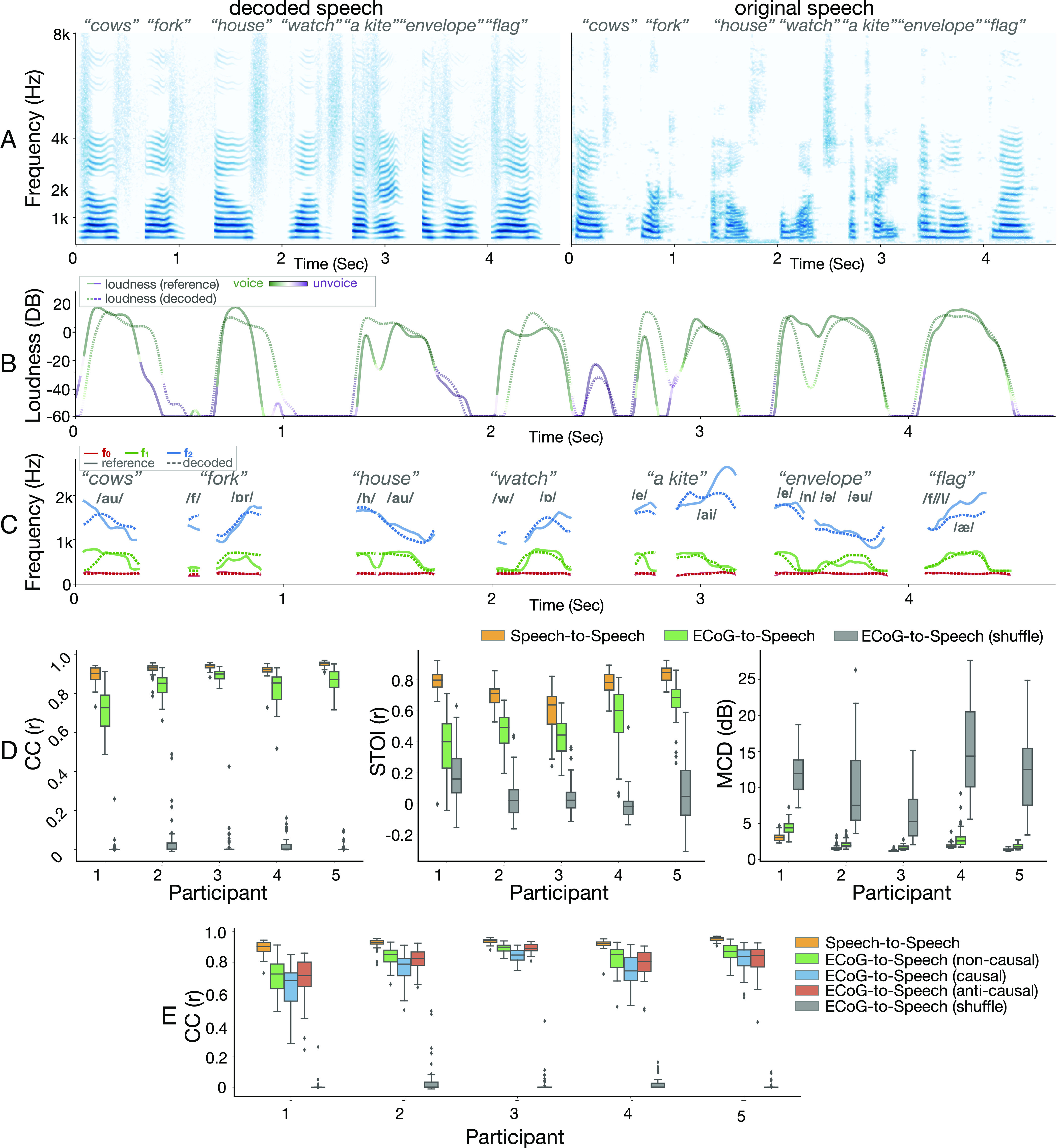
Comparison of original and decoded speech produced by the model. (*A*) Spectrograms of decoded (*Left*) and original (*Right*) speech exemplar words. (*B*) Decoded loudness parameter with the voiced (mostly vowel) or unvoiced (mostly consonant) mixing parameter color-coded over the loudness curves. The same color spread and amplitude trend between decoded (dashed) and reference (solid) curves reflect accurate decoding of voiced and unvoiced phonemes with correct energy and temporal alignment. (*C*) Frequencies of the first two formants (F1, F2) and the pitch (F0). The matching between decoded (dashed) curves and reference (solid) curves in both frequencies during each phoneme and the overall temporal dynamic leads to intelligible and naturalistic decoding of voiced sounds. (*D*) Evaluation of the decoded speech quality in objective metrics. The correlation coefficient of spectrograms (CC, *Left*), short-time objective intelligibility (STOI, *Middle*), and Mel cepstral distortion (MCD, *Right*) is used for the evaluation. Note that lower MCD values represent better performance. Both the reconstructed speech from the speech autoencoder (yellow) and the speech decoded by the ECoG decoder (green) are reported. Additionally, the performance of a model trained on shuffled data (trained by matching the decoded spectrogram from the neural signal in a given duration to a randomly selected segment of spectrograms during the entire recording session) is also reported as a control. (*E*) Comparison of the CC metric among noncausal (green), causal (blue), and anticausal (red) models. Compared to the shuffled model (the same shuffled model as in *D*), the comparable performance across noncausal, causal, and anticausal models demonstrates sufficient information for decoding speech from both feedforward and feedback signals during speech production.

We first demonstrate that our neural network approach produces accurate speech decoding with detailed acoustic features. The model’s decoded spectrogram preserves the spectro-temporal structure of the original speech. It reconstructs both vowels and consonants (Fig. 2*A*) as well as the overall spectral energy distribution (*SI Appendix*, Fig. S1). These acoustic details result in a reconstruction that preserves the speakers’ timbre (Movies S1 and S2) and leads to naturalistic voice decoding. Our model’s speech parameters which include loudness, formant frequency, and the mixing parameter (i.e., the relative weighting between voiced and unvoiced components) are decoded accurately with the correct temporal alignment of each word onset and offset (Fig. 2 *B* and *C*). The overall accuracy of the fundamental frequency (i.e., pitch), the first two modeled formants (i.e., F1 and F2), and the transition between voiced and unvoiced sounds are a major driving force for accurate speech decoding as well as naturalistic reconstruction that mimics the patient’s voice.

In order to evaluate the performance and quality of speech, we used several objective metrics, including the correlation coefficient (CC) between the decoded spectrogram and actually produced speech (23, 29, 30), an objective measure for speech intelligibility known as the Short-Time Objective Intelligibility (STOI) (23, 31), and a measure of spectral distortion, Mel-cepstral distortion (MCD) (26, 32). Across all participants and metrics, our neural decoding results performed well above chance (Fig. 2*D* in gray; estimated using shuffled data; *Materials and Methods, Visualizing Spatial Contribution Map*) and approached an upper bound of performance based on the unsupervised autoencoder (i.e., speech-to-speech) which did not use neural data. In order to verify that our model can generalize well to unseen words, we also performed stricter cross-validation providing a similar performance (*SI Appendix*, Fig. S7). The performance range across metrics and our participants were equal to and often better than the current literature (23, 26, 29, 30). Critically, all these models represent the noncausal case (Fig. 2*D*) that uses data both from the past (feedforward) and the future (feedback), as is currently a common practice (22, 23, 26, 29, 33) except a few nominal models (30).

In order to directly assess the performance of the causal (predicting using only the past) and anticausal (predicting using the future feedback) models and compare them with the noncausal (using past and future) model, which is standard in the field, we trained three separate models varying the temporal convolution direction. Our results (Fig. 2*E*) show a slight decrease in performance with the causal model. However, it performs close to the other models while providing a causal interpretation, which only uses past signals to predict future speech. This is encouraging, as it suggests that, with additional improvement in the decoder design and training, it is possible to design practically applicable neuroprosthetic speech synthesizers. Also, comparable performance between causal, anticausal, and noncausal approaches indicates a similar amount of information contained by feedforward and feedback signals.

### Feedforward and Feedback Cortical Contributions to Speech Production Are Mixed.

**Fig. 3.**
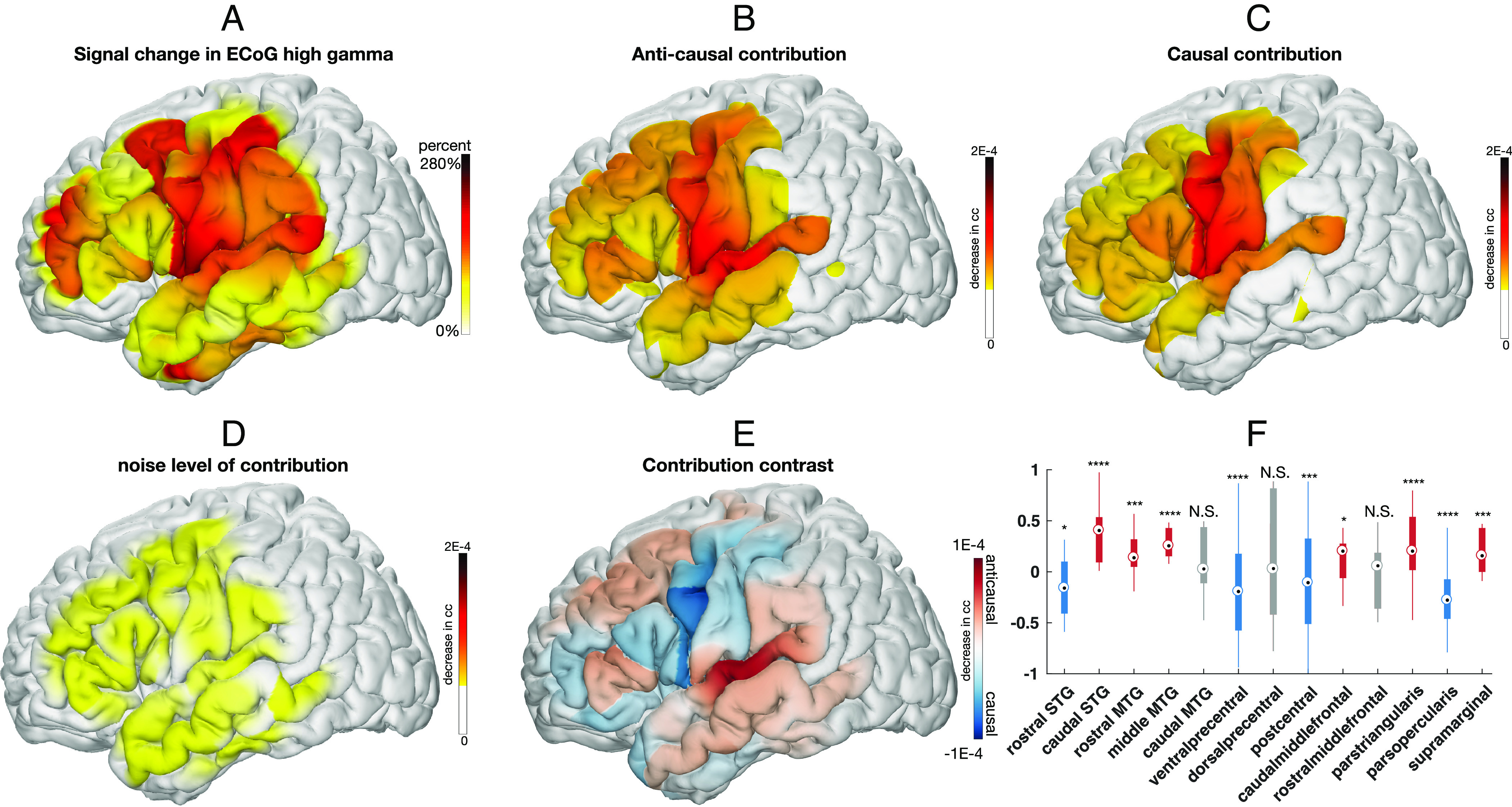
(*A*) Averaged signal of input ECoG projected on the standardized MNI anatomical map. The colors reflect the percentage change of high gamma (−250 to 750 ms relative to speech onset) during production compared to the baseline level during the prestimulus baseline period. Note that (*B*) shows the anticausal contribution of different cortical locations (red indicates higher contribution), while (*C*) illustrates the causal contribution. (*D*) The noise level of the contribution analysis was evaluated by the contributions from the shuffled model. Contributions below noise level are not shown in (*B*) and (*C*). (*E*) The contrast is obtained by taking the difference between the anticausal and causal contribution maps (red means higher anticausal contribution, while blue means higher causal contribution). The boxplots (*F*) show the average difference in each cortical region (*P-value < 0.05, **P-value < 0.01,***P-value < 0.001,****P-value < 0.0001). For purposes of visualization, we normalize each electrode by the local grid density, since our ECoG grid has hybrid density. This removes the effect of nonuniform density on the projected results (*A*–*E*).

To elucidate the feedforward and feedback contribution of different cortical regions to speech production, we examined the relative contribution of each electrode to decoding speech in our models. We derived the relative contribution by quantifying how the input signal at a particular electrode affects the overall accuracy (measured by the CC) of the reconstructed speech in the causal and anticausal models, respectively (*Materials and Methods, Visualizing Spatial–Temporal Contribution Receptive Fields*). This analysis isolates feedforward (i.e., causal) and feedback (i.e., anticausal) neural activity contributing to the decoding from the entangled ECoG neural signal, shown in Fig. 3*A*. In both the causal and anticausal models, peri-Sylvian electrodes were important for speech decoding; however, there was surprising recruitment of frontal regions when decoding speech based on the feedback (anticausal model, Fig. 3*B*) as well as recruitment of temporal sites when decoding speech based on the feedforward signals (causal model, Fig. 3*C*). We only show significant contributions that are above a threshold derived from the shuffled model (depicted in Fig. 3*D*). In order to quantify the prevalence of feedforward or feedback processing, we directly contrasted the two and projected the results onto the cortex (Fig. 3*E* and *SI Appendix*, Fig. S5). To ascertain regions that contribute significantly more to feedback or feedforward processing, we conducted a region of interest analysis based on within-subject anatomical labels of each electrode (*Materials and Methods, Electrode Localization*), testing for an increase in causal or anticausal contributions across trials (nonparametric paired Wilcoxon test; Fig. 3, as well as ANOVA controlling for the subject as a random effect; *SI Appendix*, Fig. S9). We found a surprisingly mixed distribution of causal and anticausal contributions within both temporal and frontal cortices. A majority of the temporal cortex was predominantly anticausal, including the caudal superior temporal gyrus (STG; Wilcoxon sign rank, P=1.607E−15, Z=9.6234) and portions of the middle temporal gyrus (MTG; rostral MTG: Wilcoxon sign rank test P=2.5108E−04, Z=4.9359, and middle MTG: Wilcoxon sign rank test P=1.5257E−13, Z=9.0185) as well as supramarginal cortex (Wilcoxon sign rank test P=1.1144E−04, Z=5.3919), implicating it in processing the auditory feedback signals for speech production. However, there was also a significant causal contribution in rostral STG (Wilcoxon sign rank test P=0.0332, Z=−2.9628). Similarly, the majority of the sensorimotor cortex was predominantly causal, implicating it in processing the motor speech commands, including ventral precentral (Wilcoxon sign rank, P=4.9511E−08, Z=−7.1409) and postcentral gyri (Wilcoxon sign rank, P=6.419E−04, Z=−4.9612). However, the dorsal division of the precentral gyrus was equally causal and anticausal (Wilcoxon sign rank, P=0.4349, Z=0.6525), implicating it in processing both feedforward and feedback information equally. Within the inferior frontal cortex, we found a striking division of function wherein the pars opercularis was significantly causal (Wilcoxon sign rank test, P=8.0693E−15, Z=−9.6185) while the pars triangularis was significantly anticausal (Wilcoxon sign rank test, P=2.6715E−06, Z=6.3518). Our results showed similar recruitment of the cortex when we considered the normalized causal to anticausal contribution of each individual electrode (*SI Appendix*, Fig. S2) as well as when the contribution analysis was weighted by the neural high gamma signal (*SI Appendix*, Fig. S6). Overall, these findings provide evidence for a mixed feedforward and feedback processing of speech commands and their reafference across temporal and frontal cortices, in contrast to a dichotomous view.

### Temporal Dynamics and Receptive Fields of Speech Production.

Speech production includes articulatory planning and executing the motor commands, processes that recruit distinct regions of the frontal cortex (15). However, their exact temporal receptive fields remain poorly understood. Earlier, we examined the causal and anticausal cortical contributions during speech articulation. Next, we examine articulatory planning and articulation of speech production stages and derive the related temporal receptive fields across the cortex. We leverage the receptive fields to test how cortical regions contribute differently to speech decoding with time and how frontal cortex dynamics change when feedback is introduced (after articulation starts). Both feedforward and feedback information are processed in tandem.

**Fig. 4.**
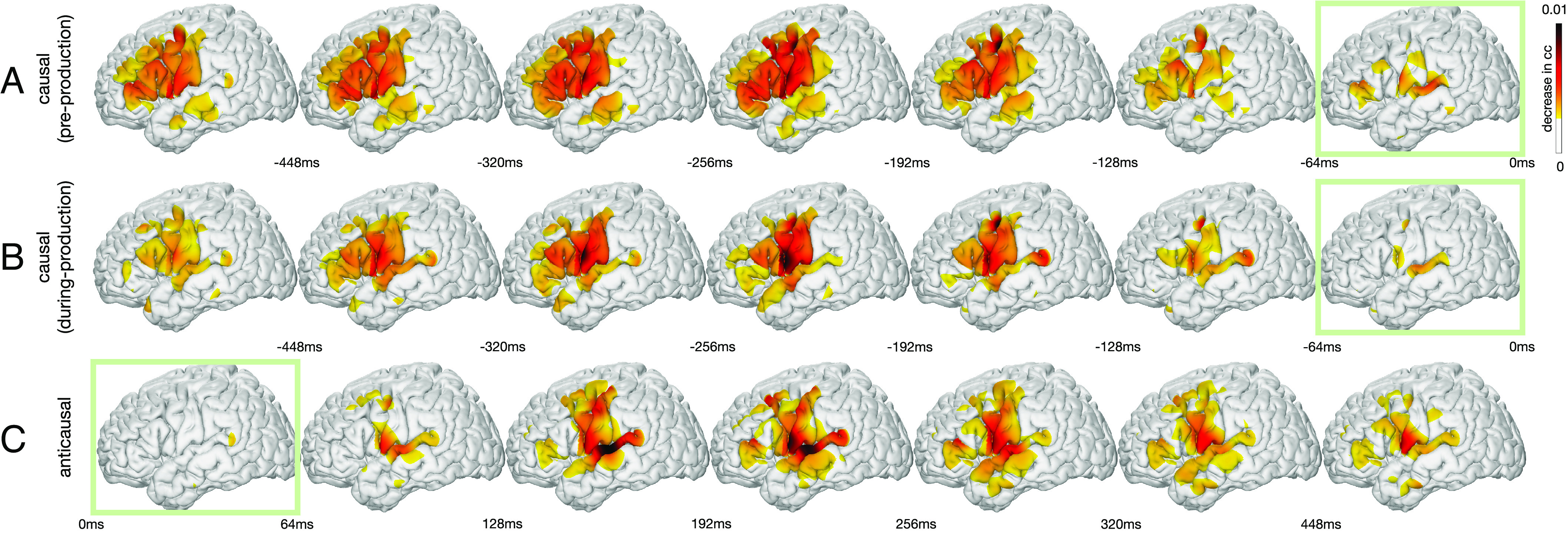
Spatial–temporal receptive fields based on decoding contribution. The contribution to speech decoding from cortical neural responses as a function of temporal delays. (*A*) The causal model is applied to preproduction data (−512 ms ∼ 0) while occluding neural data and quantifying future speech decoding across multiple delays (between neural occlusion and speech decoding). These feedforward spatial–temporal receptive fields quantify the contribution of past neural signals, prior to production, to speech decoding (negative delays). (*B*) Similarly, the causal model is applied to during-production data (0 ∼ 512 ms) representing feedforward spatial–temporal receptive fields that quantify the contribution of past neural signals (during speech production) to speech decoding (negative delays). (*C*) Representation of the feedback spatial–temporal receptive fields derived from the anticausal model that quantifies the contribution of future (positive delays) neural signals during feedback in the during-production data (0 ∼ 512 ms). These feedback receptive fields quantify the contribution of future neural signals to (past) speech decoding (positive delays). Green boxes denote the temporal window closest to zero-lag. Contributions below significance (P<0.05) representing the noise level are clipped and not shown in the plots.

We employed a similar occlusion approach to derive the temporal receptive fields as in the previous section. However, we quantified how the input signal at a particular electrode affects the accuracy of the reconstructed speech across varying delays (*Materials and Methods, Visualizing Spatial–Temporal Contribution Receptive Fields*). In brief, we employ the same trained models (Figs. 2 and 3) but occlude neural signals at different time points and quantify the change in speech decoding. We always quantify speech-decoding changes during speech; however, the neural signal occluded could be preproduction (e.g., premotor) or during-production (e.g., motor). Importantly, we quantify these contributions across all relative delays between the neural signal and speech decoding (i.e., negative in the causal direction and positive in the anticausal direction). This approach allowed us to quantify the contribution of a specific electrode in the model as a function of delay relative to speech decoding, similar to classical temporal receptive fields (i.e., TRF). We conducted this analysis for both the trained causal and anticausal models applied to data during two epochs—one prior to production (−512 ms ∼ 0 ms; Fig. 4*A*) and the other during production, which included both causal and anticausal components (0 ms ∼ 512 ms; Fig. 4 *B* and *C*). The projection of all the temporal receptive fields onto the cortex, which were significantly above a threshold derived from the shuffled model, is plotted in Fig. 4 as a function of delay. We found an increased frontal and MTG contribution prior to production (Fig. 4*A*) compared with during production (Fig. 4*B*). These processes are likely related to articulatory planning and lexical retrieval prior to speech production. During production, there was a prominent sharpening of ventral precentral gyrus receptive fields marked by a significant increase in contribution compared with preproduction (Wilcoxon sign rank test, P=8.3979E−05, Z=5.4203). While a majority of prefrontal regions engaged prior to production, there was a significant decrease in contribution across pars triangularis (Wilcoxon sign rank test, P=1.8493E−32,Z=−13.6074) and middle frontal gyri (MFG; Wilcoxon sign rank test, P=3.9177E−09, Z=−7.6103 for caudal and P=4.1581E−04, Z=−4.8311 for rostral) except for pars opercularis (Wilcoxon sign rank test, P=0.4819, Z=0.2066). Similarly, to our previous results (Fig. 3 *E* and *F*), during production, we found a significant increase in anticausal contribution for caudal STG (Wilcoxon sign rank test, P=2.6789E−17, Z=9.6711), pars triangularis (Wilcoxon sign rank test, P=0.0162, Z=3.9003), and caudal MFG (Wilcoxon sign rank test, P=0.0045, Z=3.9862) compared with causal contributions. This confirms the anatomical–functional division of the inferior and middle frontal gyri as well as caudal (Wilcoxon sign rank test, P=2.6789E−17, Z=9.6711) and rostral separation of STG (Wilcoxon sign rank test, P=0.0343, Z=−2.9457).

**Fig. 5.**
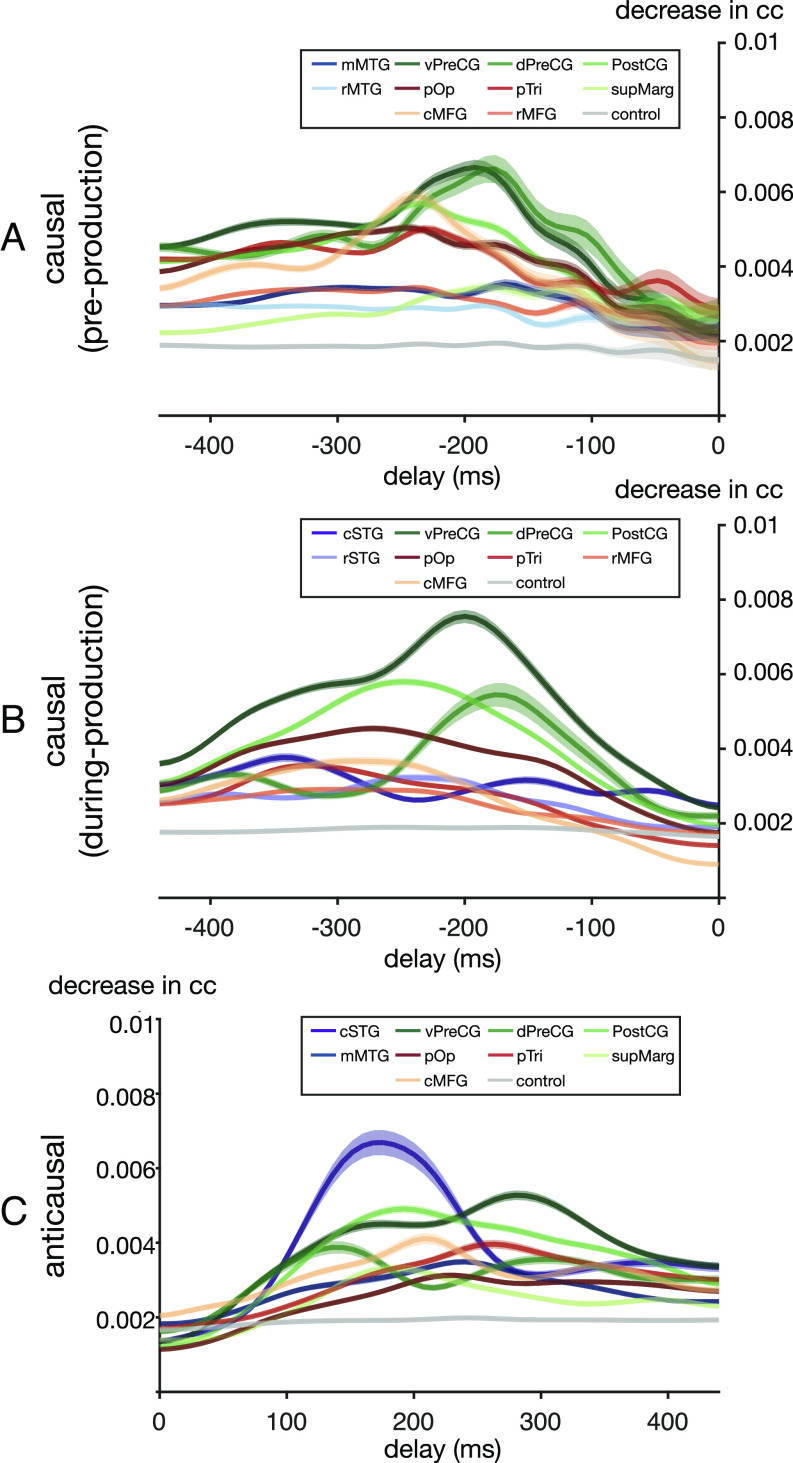
The temporal receptive field across anatomical regions. The contribution to decoding the current speech from cortical neural responses with certain temporal delays. (*A*) and (*B*) are the feedforward temporal receptive fields derived from the causal model by evaluating the contribution of past (negative delays) neural signals during a period before production onset (*A*) and after onset (*B*). (*C*) Representation of the feedback temporal receptive fields derived from the anticausal models that evaluate the contribution of future (positive delays) neural signals during feedback after articulation. The temporal propagation of the shuffled model estimates the noise level dynamics (gray curves in plots). Only regions significantly above noise level (Wilcoxon sign rank test on across-time averaged data, P<0.05) are reported.

Next, we conducted a region of interest analysis, based on within-subject anatomical labels of each electrode, in order to derive the temporal receptive curves per region (Fig. 5). This approach provides critical insight as to the temporal tuning and peak recruitment of various regions to feedforward processing prior to (Fig. 5*A*) and during production (Fig. 5*B*) as well as feedback processing (Fig. 5*C*). We found a shift in receptive field tuning for the two subdivisions of the precentral gyrus. Prior to production, dorsal and ventral precentral gyri were not significantly different from each other (Wilcoxon sign rank test, P=0.454, Z=−0.36103) and had close peak times (−196 ms, −192 ms prior to speech for ventral and dorsal precentral gyri, respectively). However, during production, these dynamics shifted and we found a significant decrease in dorsal precentral causal contribution (Wilcoxon sign rank test, P=4.7575E−05, Z=−5.6272) accompanied by temporal separation of peaks (−208 ms, −184 ms for ventral and dorsal precentral gyri, respectively; Fig. 5 *A* and *B*). Within the inferior frontal gyrus, we found that the pars opercularis was recruited similarly both prior to production and during production for feedforward processing (Wilcoxon sign rank test, P=0.5922, Z=1.7462) at a peak delay of −248 ms and −280 ms, respectively. During production, the pars triangularis had a selective increase in recruitment for anticausal compared with causal contributions (Wilcoxon sign rank test, P=0.0162, Z=3.9003), implicating it in increased feedback processing (Fig. 4*C* and *SI Appendix*, Tables S2 and S3). The anticausal receptive fields during production provide evidence for feedback processing most strongly contributed by caudal STG, with the earliest peak in contributions seen in the dorsal precentral gyrus (144 ms) and caudal STG (168 ms) followed by parietal (supramarginal 184 ms, postcentral 192 ms) and ventral precentral (280 ms) gyri (*SI Appendix*, Table S3). In order to ensure that these significant peaks were not within a temporal window possibly influenced by the autocorrelation structure of the speech or neural signal, we conducted a correlation analysis (*SI Appendix*, Fig. S11) and found that our results (Figs. 4 and 5) were outside the upper bound of signal autocorrelations (i.e., all results were greater than the near-zero correlation at 136 ms for speech spectra and 48 ms for the neural signal). Taken together, our contribution analyses suggest preferential recruitment of prefrontal cortices in feedforward processing prior to production followed by a shift in dynamics during production when feedforward and feedback signals are jointly processed with anatomical divisions of labor.

## Discussion

Our study leverages a deep learning approach together with neurosurgical recordings and dissociates direct feedforward and feedback cortical contributions during speech production. Our neural network architecture achieves exceptional decoding of speech production by tapping an interpretable compact speech representation and can be altered to focus on causal, anticausal, and noncausal decoding. Our analyses of the cortical contributions driving the performance of these models reveal a mixed distribution of feedforward and feedback processing during speech production. This was prominent in inferior, middle frontal, and superior temporal gyri which exhibited an anatomical division between feedforward and feedback processing. Last, we show a change in the temporal dynamics of prefrontal recruitment during speech planning through production, characterized by a shift of inferior frontal and precentral gyri recruitment, processing both feedforward and feedback information at different time points and spatial locations.

A growing number of studies have leveraged deep neural networks for cortical speech decoding. Convolutional neural networks (CNN) (22–25) and recurrent neural networks (RNN) (26) have mapped ECoG signals into speech and text (33, 34). However, our approach diverges from these studies. First, we develop a differentiable speech synthesizer that can generate natural speech from a compact set of interpretable speech parameters based on several signal-processing equations. This rule-based synthesizer allows for unsupervised pretraining of meaningful encoded representations (reference speech parameters) as well as reduces the capacity of the entire model and increases training data efficiency. Our speech synthesizer provides a direct mapping from the speech parameters to a participant’s voice, by using a set of participant-specific hyperparameters for the speech synthesizer that is obtained using unsupervised learning from the participant’s speech. These hyperparameters include the parameters for the prototypes of the voiced and unvoiced filters. This eliminates the need for labeled articulatory data that maps speech to articulatory dynamics as proposed by Anumanchipalli et al. (26). Second, our compact speech representation leverages an interpretable decomposition of speech into voiced and unvoiced components. This decomposition is biologically necessary and has been reported in neural representations across frontal and temporal cortices (17, 35) and stands in contrast to other traditional speech synthesizing approaches (36, 37). Last, the speech neural decoding models to date mostly employ noncausal operations. Since such decoders require both past and future information for decoding, they are not applicable to real-time speech prosthetic applications. However, a select few studies have employed causal decoding (27). Importantly, mixed operations hinder disentangling feedforward and feedback cortical contributions. In addition to providing a causal model which directly translates to practical speech prosthetics, our approach can dissociate feedforward and feedback cortical contributions during speech production.

During speech production, we process feedforward and feedback signals in tandem. Previously, the two have not been disentangled. Attempts have focused on experimental manipulations which change the feedback by shifting frequency (38) or time (39). However, these manipulations change the cortical dynamics and introduce other cognitive processes due to hearing one’s own voice altered as well as induced motor compensation. We applied convolution filters with different causality to directly train models to disentangle feedforward (i.e., causal models) and feedback (i.e., anticausal models) contributions of cortical regions. Feedforward and feedback processes are critical for driving articulatory vocal tract movement. The feedforward pathway generates an initial articulatory command and predicts sensory (auditory and somatosensory) targets; the feedback pathway compares the targets with the perceived sensory feedback and updates subsequent feedforward commands. The exact mapping between anatomical regions and their contribution to specific functional roles differ across speech motor control models (10, 11). Further, these findings have been developed based mostly on noninvasive studies which have low temporal (e.g., fMRI) or spatial resolution (e.g., M/EEG). Our high spatiotemporal resolution ECoG data together with advanced deep neural networks provide a fine-grained mapping of the cortical feedforward and feedback speech networks.

Consistent with the predominant speech motor control models, our results showed a dominant feedforward process in the ventral motor and pars opercularis of the inferior frontal gyrus, while posterior superior temporal and supramarginal gyri in the parietal lobe showed feedback (6, 11, 40). However, in contrast to these models, we found that cortices in the frontal lobe, including the pars triangularis and caudal middle frontal, are predominantly feedback in nature, while rostral STG appears feedforward. This feedback processing across frontal cortices became even stronger when we limited our analyses to the speech production epoch (Fig. 4*C* and *SI Appendix*, Table S3). Additionally, most gyri (inferior frontal, caudal middle frontal, superior temporal, precentral, and postcentral cortices; *SI Appendix*, Table S2) had both feedforward and feedback contributions above the noise level derived from the shuffled model, suggesting that feedforward and feedback processing can mix in these regions.

Our findings are likely driven by sensory-motor signaling shared across the animal kingdom, referred to as a corollary discharge (41, 42). Such a discharge from motor cortices acts to inform sensory cortices of future self-generated reafferent stimulation, often in the form of cortical suppression (19). Our peak feedforward results in the ventral precentral gyrus are consistent with such a framework (11) as well as a recent report providing direct evidence for the source of a corollary discharge in human speech (43). Interestingly, our data suggest that feedforward processing might be more distributed than previously assumed given our significant contributions across the cortex (Fig. 3*C*) as well as large causal contributions in the anterior temporal cortex (Fig. 3*E*). Further, the stark asymmetry of feedforward and feedback contributions within adjacent regions of frontal cortex (e.g., pars opercularis and pars triangularis) suggests the possibility that cortical regions are processing feedforward corollary discharge signals while also updating representations based on feedback processing.

Our results highlight the anticausal feedback signature exhibited by sensorimotor and frontal cortices. While this goes against the canonical model of the frontal cortex in an action–perception loop (44), our findings complement a growing body of evidence showing specific responses in the frontal cortex to auditory stimuli during perception. Cheung et al. (45) found distinct auditory receptive fields as well as robust passive listening responses in the ventral precentral gyrus. Similarly, the dorsal division of the precentral gyrus has recently been implicated in processing auditory feedback of altered speech as well as responding robustly during passive listening (39). However, this begs the question as to why the speech motor cortex is processing auditory information. Our feedback contribution analysis suggests that auditory processing is specifically leveraged for anticausal processing of the reafferent signals during production. Indeed, our results show that the dorsal precentral gyrus decreases feedforward processing while engaged in actual speech production (Fig. 5*B*) and is recruited for feedback at an early time point together with temporal cortices (Fig. 5*C*). Under this view, the auditory frontal responses seen during passive listening may constitute a representation dedicated to feedback processing when speech is produced.

To summarize, we provided an approach to decode speech production and interrogate the recalcitrant problem of mixed feedforward and feedback processing during speech production. We were able to leverage feedforward processing only in causal models to drive neural speech prosthetics [as opposed to the literature using noncausal processing (22, 23, 26, 29, 33)] as well as provide insights into the underpinning cortical drivers. Our results suggest a mixed cortical architecture in frontal and temporal cortices that dynamically shifts and processes both feedforward and feedback signals across the cortex. This is in contrast to previous views associating feedforward processing with primarily prefrontal and motor cortices while feedback processing is associated with the superior temporal cortex.

## Materials and Methods

### Experimental Model and Subject Details.

#### Participants and experiments.

The neural data were obtained from five patients (three male) who were native English speakers, undergoing treatment for refractory Epilepsy at NYU Langone Hospital, with implanted electrocorticographic (ECoG) subdural electrode grids. All experimental procedures were approved by the NYU Grossman School of Medicine Institutional Review Board. Patients provided written and oral consent at least one week prior to surgery by a research team member after separate consultation with the clinical care provider. The subjects were instructed to complete five tasks to pronounce the target words in response to certain auditory or visual stimuli. The subjects were asked to freely respond after the stimuli were presented without any cue or artificial delay introduced. The five tasks were

- Auditory Repetition (AR, i.e., to repeat the auditory words),
- Auditory Naming (AN, i.e., name a word based on an auditory presented definition sentence),
- Sentence Completion (SC, i.e., complete the last word of an auditorily presented sentence),
- Visual Reading (VR, i.e., read aloud visually presented word in written form), and
- Picture Naming (PN, i.e., naming a word based on a visually presented color line drawing).

Each task contained the same 50 unique target words while varying stimulus modalities (auditory, visual, etc.). Each word appeared once in the AN and SC tasks and twice in the other tasks. For participants 1–3, the five tasks included 400 trials of the produced words and the corresponding ECoG recordings. The produced speech in each trial has an average duration of 500 ms. We repeated the same five tasks twice for participant 4 and collected data from 800 trials. For participant 5, we collected 800 trials by repeating the tasks twice, and we also ran an additional AR task (200 trials) which provided 1,000 trials in total.

#### Data collection and preprocessing.

A microphone recorded the subject’s speech during the tasks and was synchronized to the clinical Neuroworks Quantum Amplifier (Natus Biomedical, Appleton, WI), which records ECoG. The recordings sampled the peri-Sylvian cortex, including STG, IFG, precentral, and postcentral gyri. The implanted ECoG array contained total of 128 electrode channels, including standard 64 clinical 8 × 8 macrocontacts (2 mm exposed, 10 mm spacing) as well as 64 additional interspersed smaller electrodes (1 mm exposed) between the macrocontacts (providing 10-mm center-to-center spacing between macrocontacts and 5-mm center-to-center spacing between micro/macro contacts, PMT corporation, Chanassen, MN). This FDA-approved array was manufactured for the study, and a member of the research team explained to patients that the additional contacts were for research purposes during consent. The ECoG arrays were implanted on the left hemisphere in all participants’ brains, and placement location was solely dictated by clinical care. More detailed illustration of the electrodes’ coverage is shown in *SI Appendix*, Fig. S3. We trained separate sets of decoding models for each participant. We randomly selected 50 out of all trials from the five tasks for testing and used the remaining data for training. The results reported are for testing data.

Each electrode sampled ECoG signals at 2,048 Hz, which was decimated to 512 Hz prior to processing. After rejecting electrodes with artifacts (i.e., line noise, poor contact with the cortex, and high amplitude shifts), we subtracted a common average reference (across all valid electrodes and time) from each individual electrode. Electrodes with interictal and epileptiform activity were removed from the analysis (note that a large number of temporal electrodes were removed from patients 4 and 5 for this reason). We then extracted the envelope of the high gamma (70–150 Hz) component from the raw signal with the Hilbert transform and further downsampled it to 125 Hz. The signal of each electrode over the silent baseline of 250 ms before the stimulus was used as the reference signal, and each electrode’s signal was normalized to the reference mean and variance (i.e., z-score).

#### Electrode localization.

Electrode localization in subject space, as well as MNI space, was based on coregistering a preoperative (no electrodes) and postoperative (with electrodes) structural MRI (in some cases, a postoperative CT was employed depending on clinical requirements) using a rigid-body transformation. Electrodes were then projected to the surface of the cortex (preoperative segmented surface) to correct for edema-induced shifts following previous procedures (46) (registration to MNI space was based on a nonlinear DARTEL algorithm). Based on the subject’s preoperative MRI, the automated FreeSurfer segmentation (Destrieux) was used for labeling within-subject anatomical locations of electrodes. The anatomical region segments can be found in *SI Appendix*, Fig. S8.

### Speech Decoder Details.

#### Speech decoding framework.

**Fig. 6.**
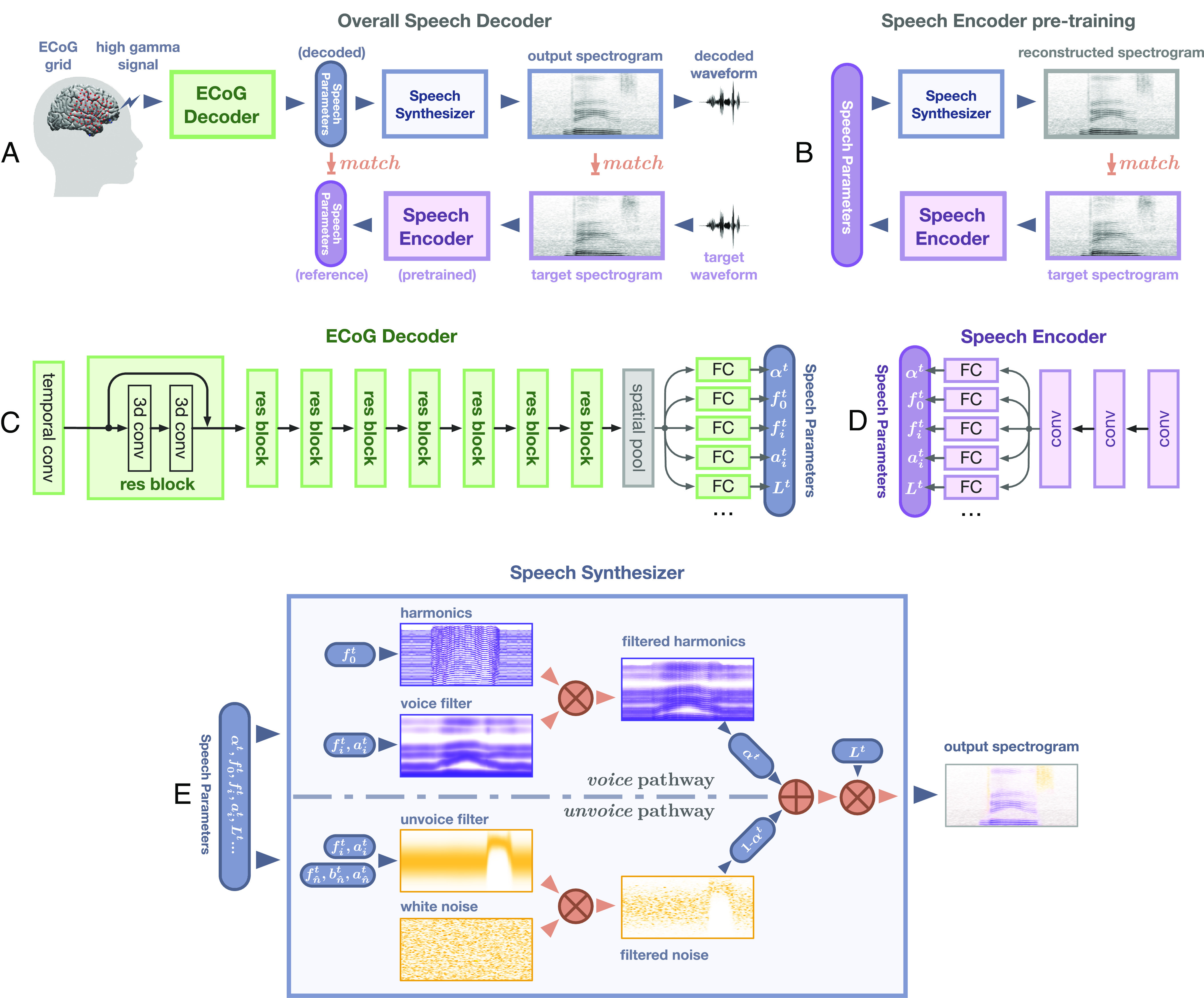
Structure of the decoding framework. (*A*) The overall network architecture. The ECoG decoder is trained to match reference speech parameters and reconstructed by a pretrained speech encoder shown in panel (*B*). (*B*) The autoencoder is used to pretrain the speech encoder. The speech encoder is trained to generate proper speech parameters that can reconstruct input spectrograms through the speech synthesizer. (*C*) The ECoG decoder is a modified spatiotemporal residual network. After an initial temporal convolutional layer and eight residual blocks (constructed by three-dimensional convolution layers), multiple subnetworks (using one or two fully connected layers) generate speech parameters separately. (*D*) The speech encoder in (*B*) has three convolutional layers followed by the same multihead output structure as in (*C*). (*E*) Illustration of the processes within the speech synthesizer. The harmonics (in voice pathway) and white noise (unvoice pathway) are generated and filtered (multiplication in spectrogram domain) by voice and unvoiced filters, respectively. The filtered results are then weighted and averaged according to the mixing parameter and then amplified by the loudness parameter. Abbreviations, FC: fully connected layer, conv: convectional layer, res block: residual block.

The backbone of our decoding framework is constructed by an ECoG decoder and a speech synthesizer (Fig. 1*A* or 6*A*). During testing, the decoder generates a set of speech parameters from the high gamma components of the neural signal, that drive a differentiable speech synthesizer to generate speech spectrograms (and corresponding waveforms by the Griffin-Lim algorithm). All models are trained on data −250 to 750 ms relative to speech onset.

- ECoG decoder. The decoder maps the ECoG signals to a set of speech parameters (describing both the voiced and unvoiced components) which are then synthesized into speech spectrograms. The ECoG decoder architecture (Fig. 6*C*) is based on recent advances in convolutional neural networks leveraging the ResNet approach (47). We construct a modified ResNet model with nine layers that treat the cortical input as a spatiotemporal three-dimensional tensor (two dimensions for the electrode array and one for time). The decoder is trained such that its output parameters match the reference parameters derived from a speech encoder (which is learned separately in an unsupervised manner). Furthermore, our approach ensures that the speech spectrogram derived from these parameters and constructed by the speech synthesizer matches the actual speech spectrogram. We use this approach to be more data-efficient and allow us to train on a small set of samples for each patient.
- Speech parameters. Our speech representation is motivated by the vocoders used for low-bit-rate speech compression dating back to the 1980s. We model speech signals as a mixture of voiced and unvoiced components, with the voiced component described by a source-filter model (dynamically filtered harmonic signals) (37) and the unvoiced component generated by white noise broadband filtering. In addition to the mixing parameter, our representation includes speech formant information (frequency, bandwidth, etc.) and loudness (i.e., the energy of speech). Overall, speech parameters are sampled at 125 Hz, and each time step involves 18 parameters to model speech. Details are found in *SI Appendix, Additional Decoding Framework Details*.
- Synthesizer. We use a set of signal processing equations (such as harmonic oscillation, noise generation, filtering, etc.) to synthesize the spectrogram from our proposed speech parameters (Fig. 6*E*). We can train the ECoG decoder with a limited amount of training data by limiting the number of speech parameters and using differentiable signal processing equations. It is noteworthy that the equations we use are differentiable (*Differentiable Speech Synthesizer* in *SI Appendix, Additional Decoding Framework Details*), which allows for backpropagation from the spectrogram to the actual learning of the decoder.
- Speech encoder. The speech encoder (Fig. 6*B*) is pretrained using an independent unsupervised approach before the ECoG decoder training. The encoder is trained to generate a set of speech parameters from a given spectrogram, from which the aforementioned speech synthesizer can reproduce the spectrogram. This pretrained encoder generates reference speech parameters from actual speech signals used for the training of the ECoG decoder. The unsupervised process can be easily used to train the speech encoder from any set of speech signals, including patient-specific speech. Importantly, this process constrains the speech parameter space to optimize the training of our ECoG decoder, and the parameters can directly drive a speech synthesizer based on differential equations.

We ruled out the possibility that our neural data suffer from a recently reported acoustic contamination (*SI Appendix*, Fig. S4) by following published approaches (48).

#### Revealing delay-dependent decoding contributions on cortex.

To quantify electrode contributions, we use an occlusion-based approach where the entire electrode is occluded and we calculate the relative change of decoding correlation coefficient (i.e., Fig. 3), or a small temporal segment of the electrode is occluded relative to speech production and we calculate the relative change of decoding correlation coefficient (i.e., Figs. 4 and 5). The temporal segment zeroed out relative to speech is varied and represents the delay in Figs. 4 and 5. Importantly, each delay represents all decoding changes during speech (at any point) and the neural signal relative to that delay (i.e., the neural signal could be prior to or during speech). This approach allows us to construct a neural receptive field of contribution as a function of relative delay compared to speech (i.e., similar to spectro-temporal receptive fields in the auditory domain and motor receptive fields in the motor domain). Before formally defining the various contribution scores, we introduce the following notations: $A_{\mathrm{ref}}\left[s\right]$: the ground truth spectrogram over a time duration S (i.e., window size) centered at time s, i.e., from s−S/2 to s+S/2, derived by the speech-to-speech autoencoder. $A_{\mathrm{intact}}\left[s\right]$: the model output with “intact” input (i.e., all ECoG signals are used). $A_{\mathrm{occlude}}^{i}\left[s|t\right]$: the model output at time duration centered at s when the ith ECoG electrode signal in the time duration centered at t from $t-\frac{T}{2}$ to $t+\frac{T}{2}$ is occluded (i.e., set to zeros). S and T define the temporal refinement of the analysis and are independent of the receptive field of the ECoG decoder (i.e., delay from temporal convolutions). r(·,·): correlation coefficient between two signals. We define the contribution of ith electrode in time duration centered at t to the output over duration centered at s by the reduction in the correlation coefficient between the output signal with the reference signal over the duration s when the ith electrode signal in duration t is occluded. Specifically,

$$\begin{array}{c} C^{i}\left[s,t\right]=\mathrm{Mean}\left\{r\left(A_{\mathrm{ref}}\left[s\right],A_{\mathrm{intact}}\left[s\right]\right)-r\left(A_{\mathrm{ref}}\left[s\right],A_{\mathrm{occlude}}^{i}\left[s|t\right]\right)\right\}, \end{array}$$

where Mean{·} denotes averaging across all testing samples.

To generate the contribution map, we first determine the contribution of each electrode (with a corresponding location in the MNI coordinate), which is then diffused into the surrounding area in the same anatomical region using a Gaussian kernel. Since our ECoG grid has hybrid density, to remove the effect of nonuniform density on the diffused result, we normalize the result of each region by the local grid density. The results shown in Figs. 3–5 are obtained by averaging the contribution maps obtained for all test samples for all participants (Figs. 4 and 5 do not include one patient due to coverage).

#### Visualizing spatial contribution map.

The contribution of the entire signal at the i-th electrode to the entire output signal, $C^{i}$, is obtained by using the method in the section above (i.e., revealing delay-dependent decoding contributions on cortex), with S and T covering the entire input and output of the signal duration. The causal and anticausal contribution plots in Fig. 3 are generated by applying this analysis to the learned anticausal model (Fig. 3*B*) and causal model (Fig. 3*C*), respectively. The contrast of the anticausal and causal contribution (Fig. 3*E*) is the difference between the causal and anticausal contribution maps. The noise level for the contribution analysis (Fig. 3*D*) is generated from the shuffled model using noncausal processing (the shuffled model is trained on an artificial dataset with temporally misaligned input–output, and thus models of different causality are equivalent). To generate the box plots per cortical region (Fig. 3*F*), we calculate the contrast contribution averaged over electrodes of the same within-subject anatomical region.

The contrast of the anticausal and causal contributions (as shown in Fig. 3*E*) for each electrode i is defined as

$$\begin{array}{c} C_{\mathrm{contrast}}^{i}=C_{\mathrm{anticausal}}^{i}-C_{\mathrm{causal}}^{i}. \end{array}$$

In order to examine the polarized electrode contributions (anticausal or causal), we calculate the normalized version of anticausal and causal contribution contrast:

$$\begin{array}{c} C_{\mathrm{polar}}^{i}=\frac{C_{\mathrm{anticausal}}^{i}-C_{\mathrm{causal}}^{i}}{C_{\mathrm{anticausal}}^{i}+C_{\mathrm{causal}}^{i}}. \end{array}$$

By normalizing the contrast of anticausal and causal contribution, $C_{\mathrm{polar}}^{i}$ emphasizes the directionality of anticausal or causal, rather than their absolute contrast. This is visualized in *SI Appendix*, Fig. S2 *A* and *B* [only electrodes with either anticausal contribution attribute ($C_{\mathrm{anticausal}}^{i}$) or causal contribution ($C_{\mathrm{causal}}^{i}$) above the noise level determined by the shuffled model].

#### Visualizing spatial–temporal contribution receptive fields.

When evaluating the contribution over a finite duration (temporal occlusion), we use small temporal scope S=T=64ms (i.e., this represents the size of the neural signal occluded, T, as well as the size of speech decoding assessed, S). To evaluate the contribution of an electrode signal to the output with various delays, denoted by τ, we average $C^{i}\left[s,s+\tau \right]$ for all s in a certain duration (from time $s_{0}$ to $s_{1}$) leading to

$$\begin{array}{c} {\tilde{\;\text{C}}}^{i}\left(\tau \right)=\frac{1}{s_{1}-s_{0}}\sum_{s=s_{0}}^{s_{1}}C^{i}\left[s,s+\tau \right]. \end{array}$$

Here, we assume that the effect of the delay is independent of the actual time of speech s. Negative τ values denote neural signals prior to a specific speech segment (s) and thus represent the causal direction. In other words, when τ≤0, ${\tilde{\;\text{C}}}_{\mathrm{causal}}^{i}\left(\tau \right)$ reveals the causal contribution of electrode i to the speech output at the different delays (Fig. 4 *A* and *B*). Conversely, positive τ values denote neural signals after a specific speech segment (s) and thus represent the anticausal direction (i.e., τ≥0, ${\tilde{\;\text{C}}}_{\mathrm{anticausal}}^{i}\left(\tau \right)$ reveals the anticausal contribution shown in Fig. 4*C*). Causal neural contributions to speech can originate prior to speech onset (prearticulation) or during speech production (during-articulation). To investigate the prearticulation contribution, we restrict s+τ to the prearticulation period so that neural signals are only occluded before speech onset. Similarly, to investigate during-articulation contribution, we restrict s+τ to postspeech onset. Importantly, speech decoding (s) is always assessed during speech, while the neural signals are occluded either prearticulation or during-articulation time periods. This occlusion analysis can be performed prearticulation for the causal model only (past neural signals prior to speech onset contributing to future speech decoding) and during-articulation for both the causal model (past neural signals during speech production contributing to future speech decoding) and anticausal model (future neural signals during speech production contributing to past speech decoding) shown in Fig. 4.

#### Visualizing per region temporal contribution receptive field.

Similarly to the per-region plot in Fig. 3*F*, we average the spatial–temporal receptive field data (Fig. 4) over within-subject anatomical region labels to generate a temporal contribution curve for each region (Fig. 5). The control curve is generated by applying the same method for the shuffled model (gray curves in Fig. 4). We omit curves that are not significantly above noise level by Wilcoxon sign rank testing between averaged (over time) region contribution curves and the averaged (over time) noise level curve (*SI Appendix*, Table S2). In order to calculate peak contributions, we smooth the curves using an 88-ms Hann window and take the maximal value. The motivation of smoothing the temporal curves was to provide unique estimates for peak contribution (i.e., maximum). The smoothed curves are plotted in Fig. 5, and the original unsmoothed, raw, data show the same dynamics and are plotted in *SI Appendix*, Fig. S10.

### Additional Decoding Framework Details.

#### Differentiable speech synthesizer.

In a traditional vocoder, speech is generated by switching between voiced and unvoiced content. Each content comes from an autoregressive system driven by a certain excitation signal that is either a harmonic signal or a white noise signal (49). Inspired by such a process, we construct our speech synthesizer shown in Fig. 6. It consists of two pathways. The voice pathway generates a voiced component by driving a harmonic excitation with time-varying fundamental frequency (i.e., pitch) $f_{0}^{t}$ through a voiced filter consisting of N formant filters, each described by a center frequency $f_{i}^{t}$ and an amplitude $a_{i}^{t},i=1,2,...,N$. Each formant filter can be viewed as an IIR filter defined explicitly in the frequency domain and is applied to the excitation signal (spectrogram) by a frequency-wise multiplication. Note that we parameterize the bandwidth $b_{i}^{t}$ as a function of the center frequency $f_{i}^{t}$. The unvoice pathway generates an unvoiced component by driving a white noise through an unvoiced filter described as a center frequency $f_{n}^{t}$, bandwidth $b_{n}^{t}$ and amplitude $a_{n}^{t}$ (in addition to the N formant filters for the voice pathway). These two components are adaptively combined with a time-varying mixing factor $\alpha^{t}$, controlling the relative contribution between voiced sounds (for sonorant phonemes including vowels and nasals) and unvoiced sounds (for voiceless plosives and fricatives, such as /p/ and /s/, respectively). The voiced plosives and fricatives (such as /b/ and /z/, respectively) can be generated as a combination of voiced and unvoiced components. Finally, the combined signal is amplified by a loudness parameter $L_{t}$. In our study, we used N=6 formants. The synthesizer is driven by a total of 18 time-varying speech parameters, including the fundamental (or pitch) frequency $f_{0}^{t}$, the mixing factor between the two pathways $\alpha^{t}$, the 12 parameters for the voiced filter ($f_{i}^{t},a_{i}^{t}$) and the three parameters for the unvoiced filter $f_{n}^{t}$, $b_{n}^{t}$, $a_{n}^{t}$, and the loudness $L^{t}$. Given the parameter values at each time sample, the synthesizer can generate a spectrogram sample. The spectrogram is a differentiable function of the speech parameters so that we can backpropagate the gradient of the training loss in terms of the predicted spectrogram to the speech parameters, which can then be backpropagated to either the speech encoder or the ECoG decoder parameters. Specifically, let the $V^{t}\left(f\right)$ represent the spectrogram of the voicing component, $U^{t}\left(f\right)$ that of the unvoicing component, and $\alpha^{t}\in \left[0,1\right]$ the mixing factor. The combined spectrogram can be written as $S^{t}\left(f\right)=\alpha^{t}V^{t}\left(f\right)+\left(1-\alpha^{t}\right)U^{t}\left(f\right)$. Finally, the synthesized speech spectrogram is ${\tilde{S}}^{t}\left(f\right)=L^{t}S^{t}\left(f\right)$, where $L^{t}$ is the loudness that modulates the signal cross-time.

##### Formant filters in the voice pathway.

The filter in the voice pathway consists of multiple formant filters, corresponding to the multiple formants associated with vowels. The formant filter shape over frequency, which is related to the resonance property of the vocal tract, is closely related to the timbre of speakers’ voice (50). We have found that a predefined analytic form such as generalized Gaussian cannot cover all feasible filter shapes. Instead, we learn a speaker-dependent prototype filter for each formant based on the speaker’s natural speech. We represent the prototype filter $G_{i}\left(f\right)$ for the i-th formant as a piecewise linear function, linearly interpolated from $g^{i}\left[m\right],m=1...M$, the amplitudes of the filter at M uniformly sampled frequencies up to $f_{\max}$. We restrict the resulting filter $G_{i}\left(f\right)$ to be unimodal (with a single peak of value 1) by properly constraining g[m]. Given g[m],m=1...M, the peak frequency $f_{i}^{\mathrm{proto}}$ and the half-power bandwidth $b_{i}^{\mathrm{proto}}$ can be determined. The actual formant filter at any time can be written as a shifted and scaled version of $G_{i}\left(f\right)$. Specifically, at time t, given an amplitude ($a_{i}^{t}$), a center frequency ($f_{i}^{t}$), and a bandwidth ($b_{i}^{t}$), the i-th formant filter is given by

[1] $$\begin{array}{c} F_{i}^{t}\left(f\right)=a_{i}^{t}\cdot G_{i}\left(\frac{b_{i}^{\mathrm{proto}}}{b_{i}^{t}}\cdot \left(f-f_{i}^{t}\right)+f_{i}^{\mathrm{proto}}\right). \end{array}$$

Then, the filter for the voice pathway with N formant filters can be written as $F_{h}^{t}\left(f\right)=\sum_{i=1}^{N}F_{i}^{t}\left(f\right)$. We learn the parameters g[m],m=1...M for $G_{i}\left(f\right)$ during the unsupervised pretraining of the speech encoder, which does not require neural data. Fitting such a prototype filter is not data-hungry even with a relatively large M. We used M=20 in our experiment. Although two formants (N=2) have been shown to suffice for intelligible reconstruction (7), we use N=6 in our experiments for more accurate synthesis. We denote the parameter set for the voiced filter at time t by $S^{t}=\left\{\left(f_{i}^{t},a_{i}^{t},b_{i}^{t}\right)|i\in \left\{1,\cdots ,N\right\}\right\}$. The bandwidth $b_{i}^{t}$ parameters are not independent speech parameters, rather functions of the center frequencies $f_{i}^{t}$.

##### Unvoiced filter.

For the unvoice pathway, we add a broadband filter described by $\left\{\left(f_{\hat{n}}^{t},a_{\hat{n}}^{t},b_{\hat{n}}^{t}\right)\right\}$. The shape of this filter $F_{\hat{n}}^{t}\left(f\right)$ follows Eq. **1** but with the filter coefficients $(\alpha_{i}^{t}$, $f_{i}^{t}$, $b_{i}^{t})$ replaced by $(\alpha_{\hat{n}}^{t}$, $f_{\hat{n}}^{t}$, $b_{\hat{n}}^{t})$. The bandwidth is constrained to satisfy $b_{\hat{n}}^{t}\;>\;$2,000Hz, following the broadband nature of obstruent phonemes. We also keep the multiple formant filters in the voiced filter described by $S^{t}$. This is motivated by the fact that human beings differentiate consonants with similar sounds such as /p/ and /d/, not only by the immediate burst of these sounds but also the development of the following formant frequency until the next vowel (51). To encode such formant transitions, we use the same formant filter parameters for modeling the narrow bandpass in both the voiced component and the unvoiced component. The parameter set for the unvoiced component is thus $T^{t}=S^{t}\cup \left\{\left(f_{\hat{n}}^{t},a_{\hat{n}}^{t},b_{\hat{n}}^{t}\right)\right\}$. The overall filter for the unvoice pathway is $F_{n}^{t}\left(f\right)=F_{\hat{n}}^{t}\left(f\right)+\sum_{i=1}^{N}F_{i}^{t}\left(f\right)$.

To further reduce the parameter space dimension, we model the bandwidth $b_{i}^{t}$ of a formant filter as a piecewise linear function of the center frequency $f_{i}^{t}$. We assume

$$b_{i}^{t}=\left\{\begin{array}{cc} a\left(f_{i}^{t}-f_{\theta }\right)+b_{0}, & \;\text{if}f_{i}^{t}>f_{\theta }\\ b_{0}, & \;\text{otherwise}, \end{array}\right.$$

where threshold frequency $f_{\theta }$, slope a, and baseline bandwidth $b_{0}$ are three parameters that can be learned during unsupervised pretraining, shared among all formant filters.

##### Harmonic excitation.

In the voice pathway, the voiced filter is applied on the harmonic excitation. This pathway models the human production of vowels and nasals, which results from the voice excited by the vocal cord shaped by the vocal tract. The excitation is constructed by sinusoidal harmonic oscillations with a time-varying fundamental frequency $f_{0}^{t}$. Inspired by the formulation in ref. 52, we define the harmonic excitation $h^{t}$ as $h^{t}=\sum_{k=1}^{K}h_{k}^{t}$, where K is the total number of harmonics (K=80 in our experiment). Assuming the initial phase is 0, each harmonic resonance $h_{k}^{t}$ at time step t has an instant phase that is the accumulation of resonance frequency in the past. Specifically, the k-th resonance at time step t is $h_{k}^{t}=\sin(2\pi \sum_{\tau =0}^{t}f_{k}^{\left(\tau \right)})$, where $f_{k}^{\left(t\right)}=kf_{0}^{\left(t\right)}$. Denoting the spectrogram of $h^{t}$ as $H^{t}\left(f\right)$, the spectrogram of the voiced component is the multiplication of $H^{t}\left(f\right)$ and the voiced filter, i.e., $V^{t}\left(f\right)=H^{t}\left(f\right)F_{h}^{t}\left(f\right)$.

##### Noise excitation.

The unvoiced pathway models consonants like plosives and fricatives, where the vocal tract and human mouth filter the airflow through the mouth. It follows a similar process as in the harmonic counterpart. The major difference is that the excitation being filtered becomes stationary white Gaussian distributed noise $\hat{n}\left(t\right)∼N\left(0,1\right)$, with a corresponding spectrogram $N^{t}\left(f\right)$. The filtered noise spectrogram (i.e., the unvoiced component) is $U^{t}\left(f\right)=N^{t}\left(f\right)F_{n}^{t}\left(f\right)$.

#### ECoG decoder and speech encoder.

The ECoG decoder is constructed by a three-dimensional ResNet that treats time-varying signals on an ECoG grid array as spatiotemporal three-dimensional tensors (width × height × time duration). As is depicted in Fig. 6*C*, after an initial temporal convolutional layer [with 128 output features, each corresponding to a convolution kernel of size 1×1×9 (72 ms)], the signal passes through eight residual blocks. Each block contains two three-dimensional convolutional layers [with 128 output features, each corresponding to a convolution kernel of size 3×3×5 (40 ms)]. The output of the residual blocks creates a shared latent representation consisting of 128 features (each is a one-dimensional temporal signal by average pooling the two spatial dimensions), which is then fed into different output heads (each applies each consists of one or two fully connected layers acting on the 128 features at the same time point) to generate speech parameters. The overall temporal receptive field for generating one speech parameter sample is 73 temporal samples of 584 ms.

The speech encoder network architecture we choose is as simple as possible to demonstrate the effectiveness of the speech synthesizer design. We use three layers of temporal convolution (we treat the frequency axis of the spectrogram as the feature dimension) to generate a latent representation (Fig. 6*D*). Each convolutional layer has 128 output features and a temporal kernel size of three frames (24 ms). To output the speech parameter, we apply the same multihead structure to the latent representation as in the last layer of the ECoG decoder.

In order to implement models with different temporal causality, each convolutional layer of the speech encoder and ECoG decoder models were implemented as either causal, anticausal, or noncausal corresponding to the model causality.

#### Loss and training hyperparameters.

The speech encoder is trained with a weighted average of the mixed spectral and parameter loss. The mixed spectral loss (52) is defined as

$$\begin{array}{c} L_{\mathrm{MSS}}\left({\tilde{S}}^{t}\left(f\right),S^{t}\left(f\right)\right)=L_{\;\text{lin}}\left({\tilde{S}}^{t}\left(f\right),S^{t}\left(f\right)\right)+L_{\;\text{mel}}\left({\tilde{S}}^{t}\left(f\right),S^{t}\left(f\right)\right), \end{array}$$

in which,

$$\begin{array}{cc} L_{\;\text{lin}}\left(x,y\right) & ={∥x-y∥}_{1}+{∥\;\text{log}x-\;\text{log}y∥}_{1}\\ L_{\;\text{mel}}\left(x,y\right) & ={∥x_{\text{mel}}-y_{\text{mel}}∥}_{1}+{∥\;\text{log}x_{\text{mel}}-\;\text{log}y_{\text{mel}}∥}_{1}, \end{array}$$

where $S^{t}\left(f\right)$ and ${\tilde{S}}^{t}\left(f\right)$ denote the ground truth and reconstructed spectrograms, respectively, subscript lin means that the frequency is in the linear scale, while the subscript mel means the frequency is in the mel scale. In our experiments, we use 256 frequency samples (ranging from 0 to 8,000 Hz) for both linear and mel scale speech spectrograms.

Let us denote the j-th reconstructed speech parameter as ${\tilde{P}}_{j}^{t}$ and its reference $P_{j}^{t}$; the overall training loss for the ECoG decoder becomes

$$\begin{array}{cc} L & =L_{\mathrm{spectrogram}}+L_{\mathrm{speech}\mathrm{parameters}}\\ & =\lambda_{0}L_{\mathrm{MSS}}\left({\tilde{S}}^{t}\left(f\right),S^{t}\left(f\right)\right)+\sum_{j}\lambda_{j}({∥{\tilde{P}}_{j}^{t}-P_{j}^{t}∥}_{2}^{2}), \end{array}$$

where $\lambda_{j}$ balance the contribution from different loss terms since they have different physical meanings and scales.

Both the speech encoder and ECoG decoder are fitted by the Adam optimizer with hyperparameters: $lr=10^{-3},\beta_{1}=0.9,\beta_{2}=0.999$. We train an individual ECoG decoder and speech encoder per patient. The pretraining of the speech encoder and the training of the ECoG decoder share the same training/testing set partition.

### Quantification and Statistical Analysis.

We perform most of our statistical tests with Wilcoxon sign rank. The one-way ANOVA test is used to confirm the causal and anticausal contributions per-region with the subject treated as a random variable. Prior to the ANOVA test, we first verified that causal and anticausal follow a normal distribution (using a Kolmogorov–Smirnov test). Statistical significance is indicated as *P-val < 0.05, **P-val < 10${}^{-2}$, ***P-val < 10${}^{-3}$

## Supplementary Material

Appendix 01 (PDF)

Movie S1. Decoded samples (original speech followed by decoded speech).

Movie S2. Decoded samples (decoded speech followed by original speech).

## Data Availability

Some study data available (the code and data reported in this paper will be shared by the lead contact upon request, due to the sensitivity of patient data and ability to anonymize all data. Code to generate the results can be found at https://github.com/flinkerlab/DistributedFeedforwardFeedbackProcessing) (53).
