# Supplementary material for: Distributed feedforward and feedback cortical processing supports human speech production
Source: Proc Natl Acad Sci U S A. 2023 Oct 11;120(42):e2300255120. doi: 10.1073/pnas.2300255120 (PMC10589651; doi:10.1073/pnas.2300255120)
Supplement: Supplementary file 1 — Appendix 01 (PDF) [file pnas.2300255120.sapp.pdf]

PNAS

Supporting Information for

Distributed Feedforward and Feedback Cortical Processing Supports Human Speech Production

Ran Wang, Xupeng Chen, Amirhossein Khalilian-Gourtani, Leyao Yu, Patricia Dugan, Daniel Friedman, Werner Doyle, Orrin Devinsky, Yao Wang, and Adeen Flinker

Corresponding Author: Adeen Flinker.
E-mail: adeen.flinker@nyulangone.org

This PDF file includes:

Figs. S1 to S11
Tables S1 to S5
Legends for Movies S1 to S2
SI References

Other supporting materials for this manuscript include the following:

Movies S1 to S2

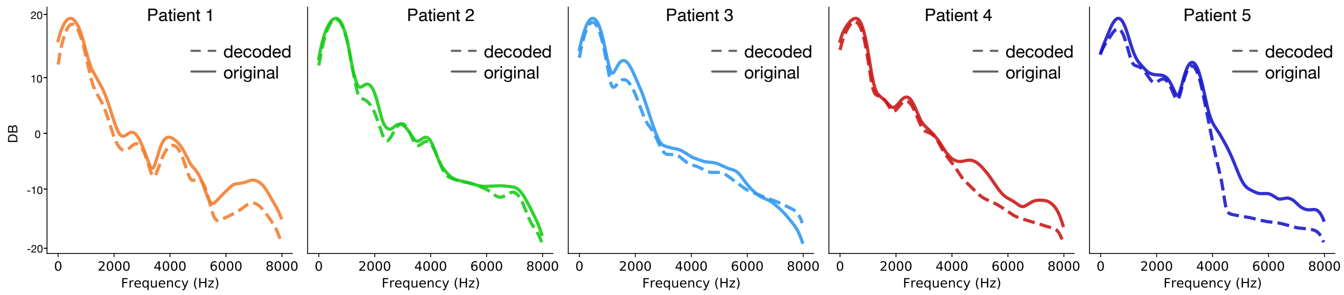

Fig. S1. The spectral energy distribution of the decoded and original speech for five patients. Visualized by averaging the broadband spectrogram magnitude across time of all test samples. **Related to: Figure 1,2,6**

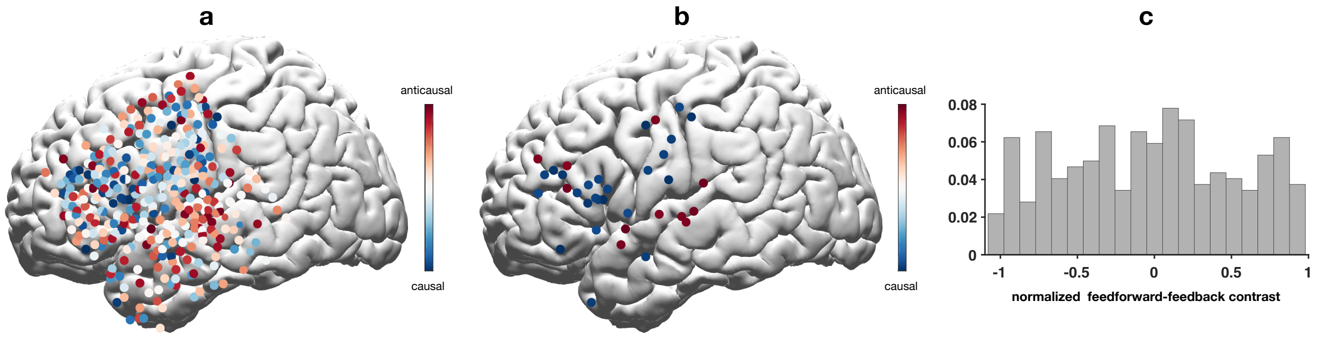

Fig. S2. Normalized contrast of feedforward vs. feedback contribution. (a) Electrode level feedforward-feedback contribution contrast, normalized by the feedforward and feedback contribution magnitude sum. (b) Electrodes with largest feedforward-feedback polarity quantified as a normalized contrast magnitude greater than 0.9. (c) The histogram of the normalized contrast. Positive bins correspond to the anti-causal direction. **Related to Figure 3**

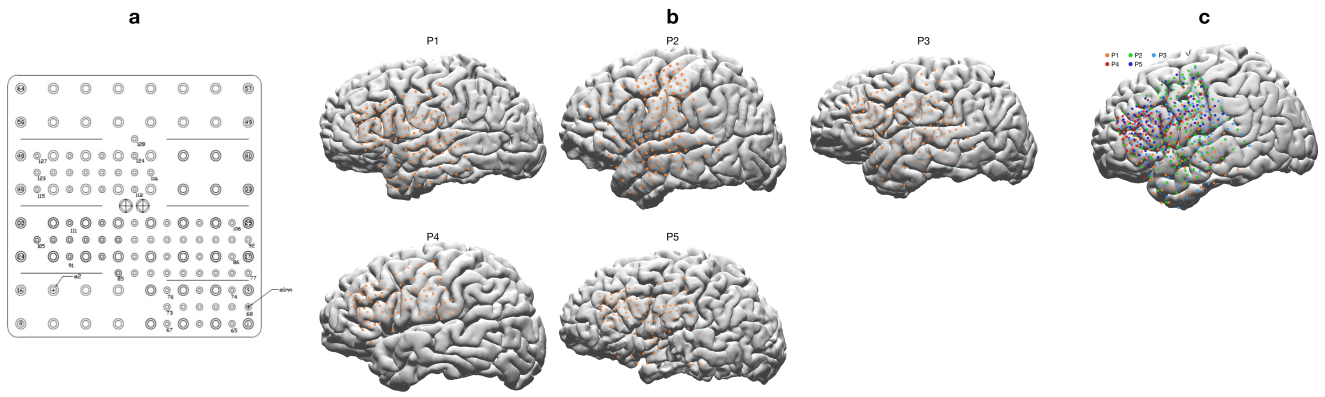

Fig. S3. Electrode array and implant location of all five patients (P1-P5) in our experiments. (a) Schematic of the 128 electrode hybrid density ECoG array. (b) Electrodes on cortex of each subject. (c) Electrodes from all subjects in the MNI coordinate system. **Related to Figure 3,4**

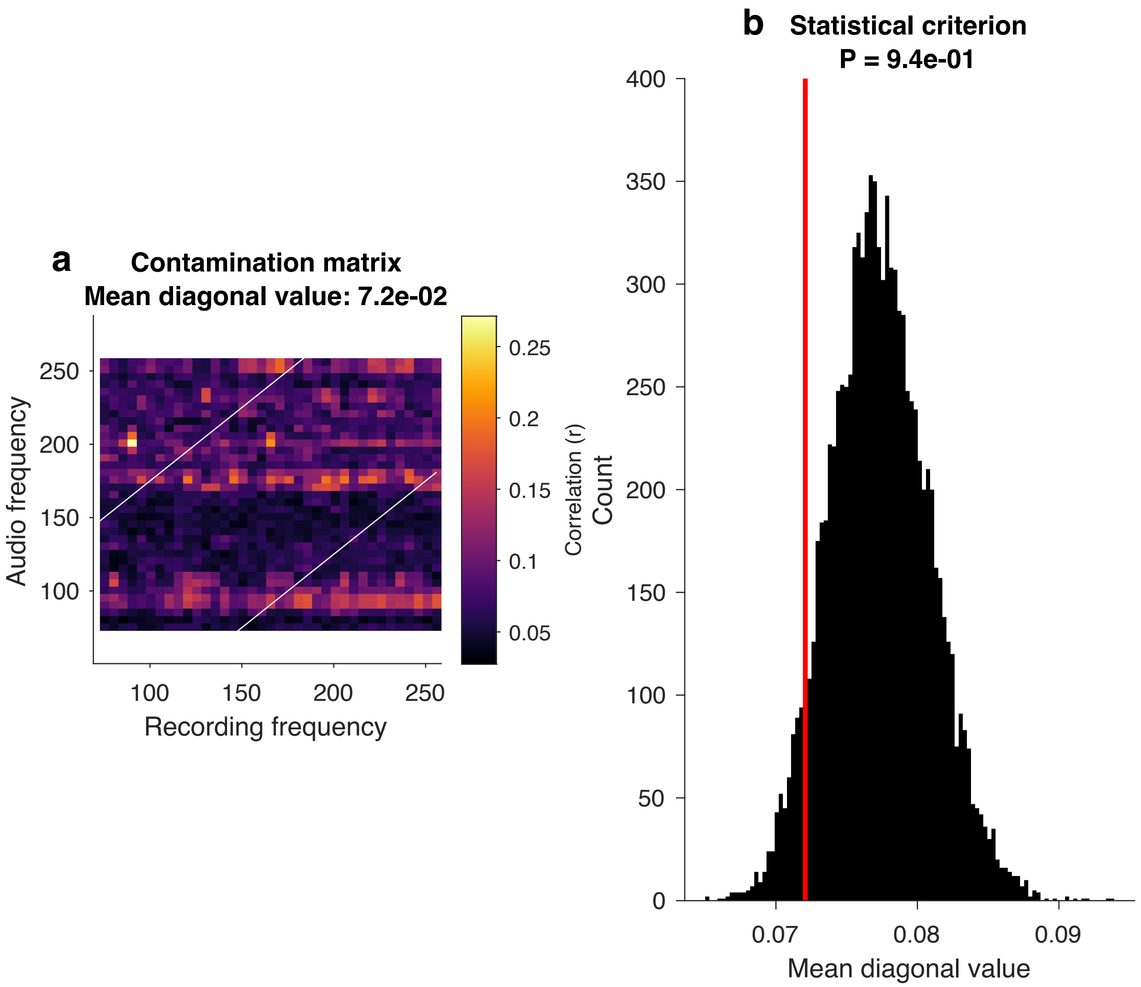

Fig. S4. Acoustic contamination evaluation. (a) The correlation matrix between frequency components in the audio signal and the neural ECoG recordings. (b) Statistical assessment of contamination. The mean of the diagonal of the contamination matrix (the vertical rad bar) and histogram of such value in 10000 shuffled contamination matrices. The criterion (P$= 9.4E - 1$) of ECoG recordings which rejects the null hypothesis that neural recordings have acoustic contamination. The analysis follows the protocol reported in (1). **Related to Figure 2**

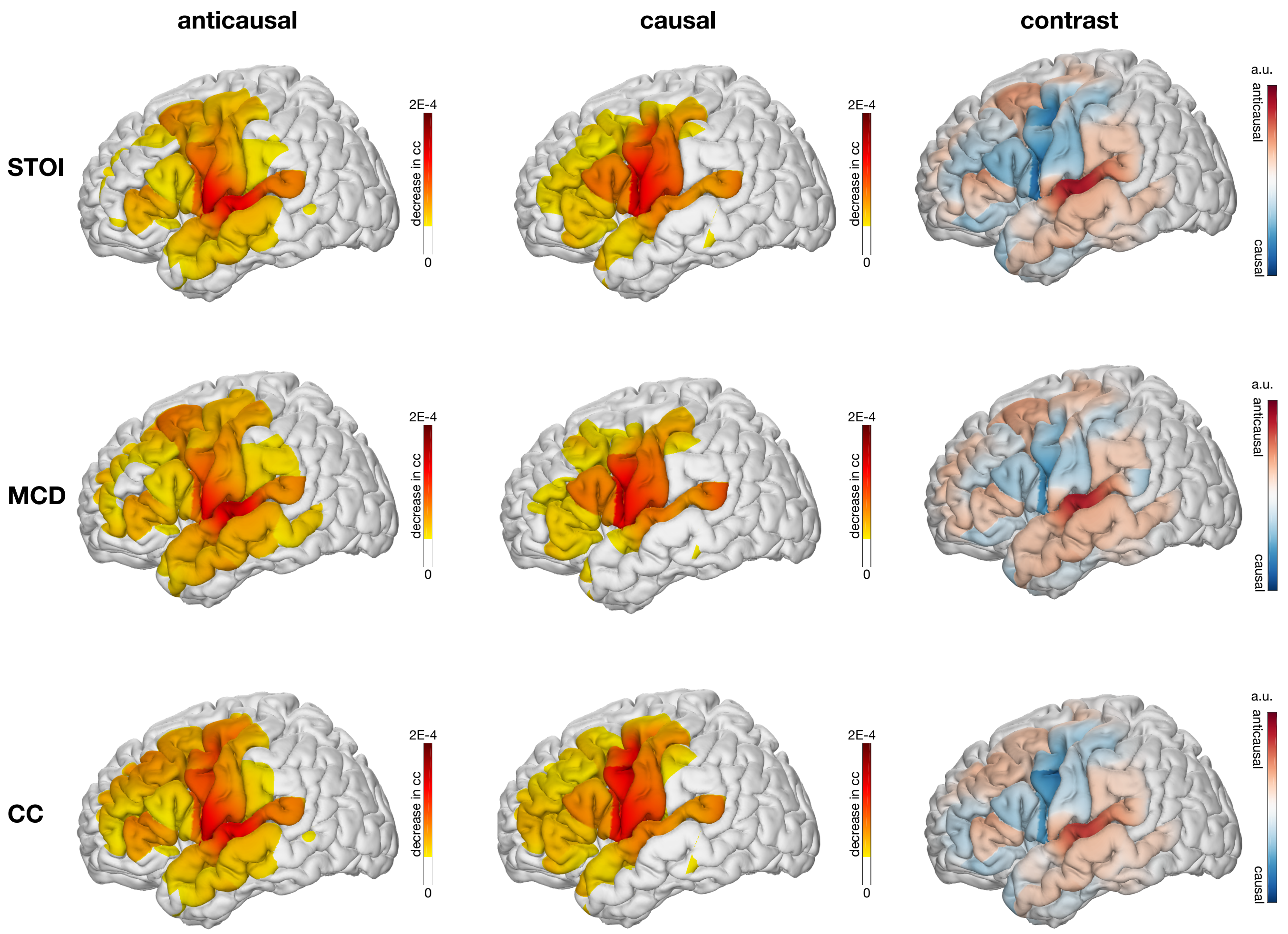

Fig. S5. Contribution analysis in terms of speech intelligibility (STOI) and voice quality (MCD). Relative electrode contribution for the anti-causal and causal models is shown, as well as the contrast between the two. The models were assessed separately using a correlation coefficient (CC) as well as a voice quality metric (MCD), and an intelligibility metric (STOI). Overall, the contribution maps for each causality model and their contrast across different metrics are similar. However, increased causal contribution in STOI compared with MCD can be seen over sensorimotor cortices as well as anti-causal contribution over MFG. **Related to Figure 3**

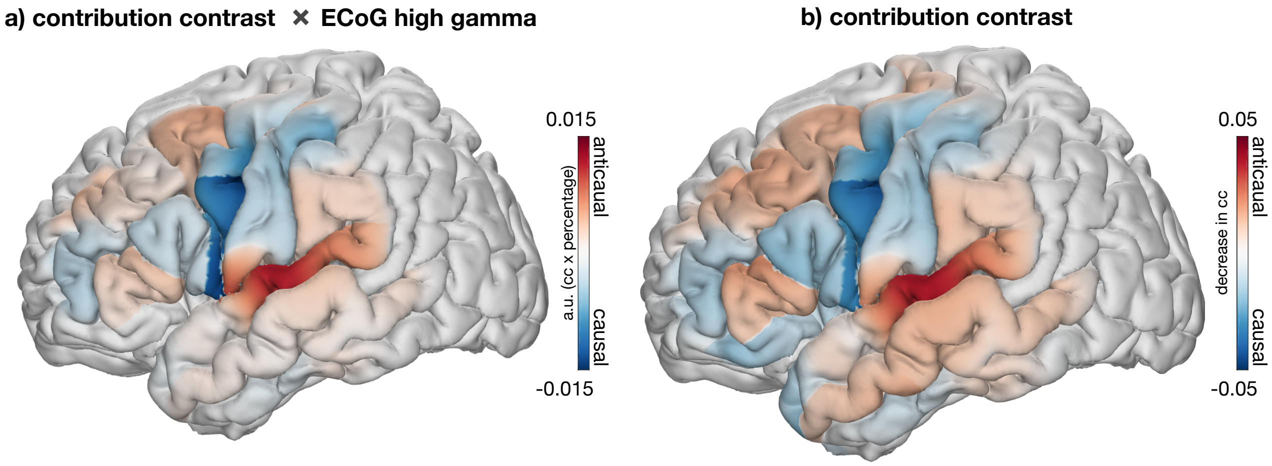

Fig. S6. Contribution contrast between causal and anti-causal models. a) Contribution contrast weighted by the ECoG signal change. b) Contribution contrast alone. **Related to: Figure 3,4**

.

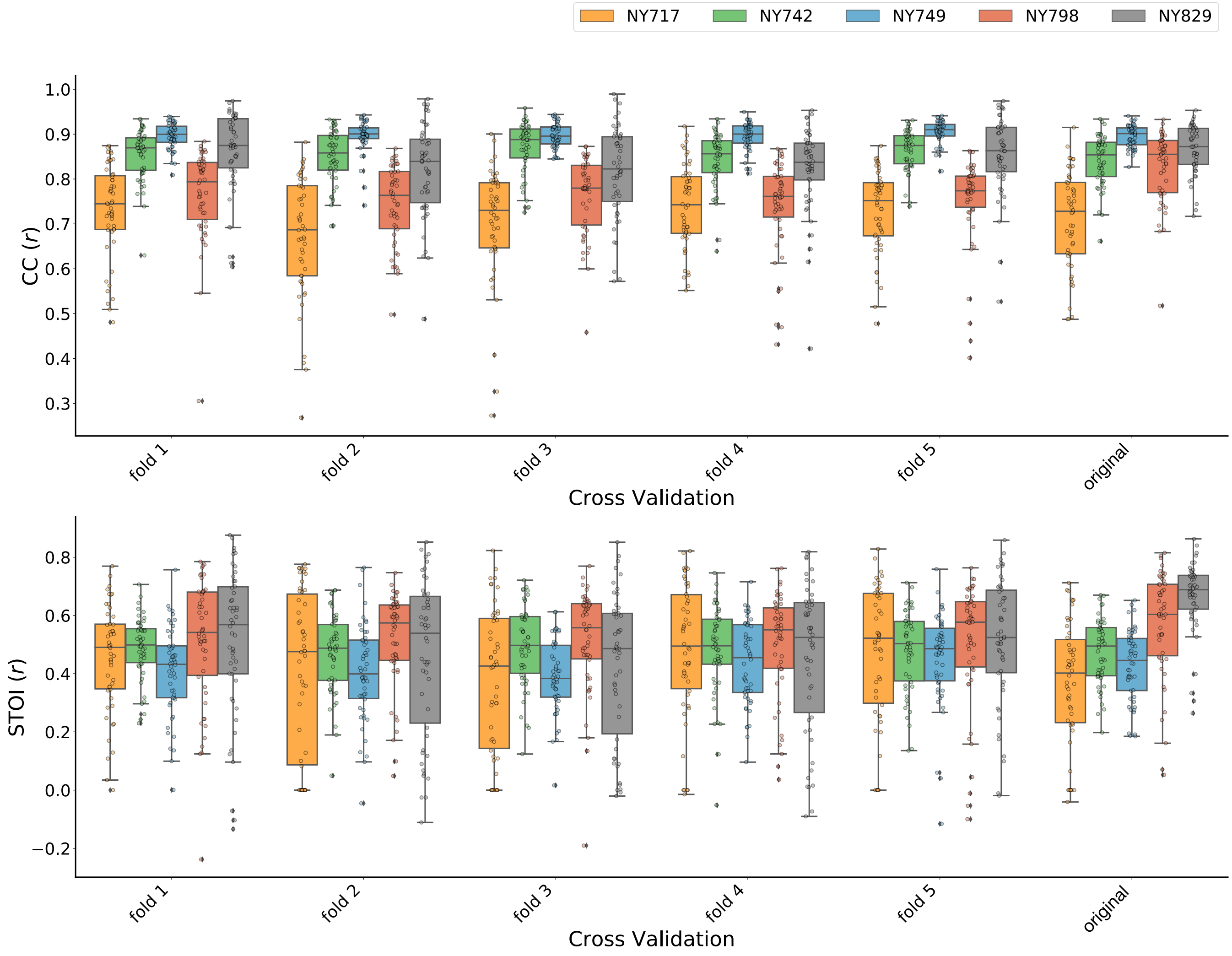

Fig. S7. Decoding performance in a 5-fold cross-validation approach for both CC and STOI metrics. In Figure 2, we held out the randomly chosen testing data during training. However, the same word could have appeared in both the training and testing sets (while the actual trials differed). In order to verify that our model can generalize well to unseen words, we performed a stricter cross-validation wherein each fold, we randomly removed 10 unique words from the training set for the training of the ECoG decoder, audio encoder, and speech synthesizer. The resulting models are tested on the held-out target words, and the model performance per patient and fold are depicted. "Original" refers to the validation approach shown in Figure 2. **Related to Figure 2**

.

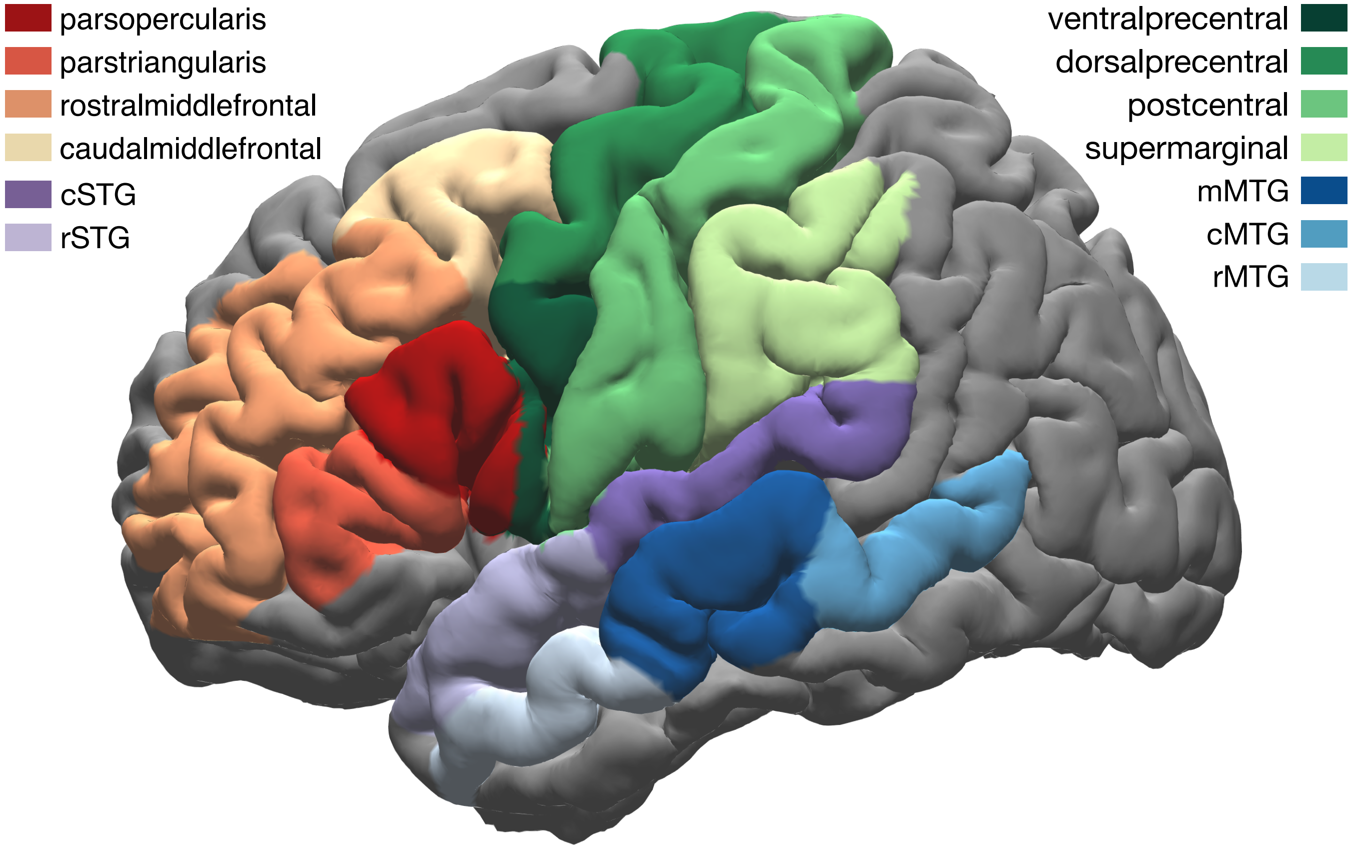

Fig. S8. Anatomical region segmentation in the MNI space used in this study. **Related to: Figure 3,5**

.

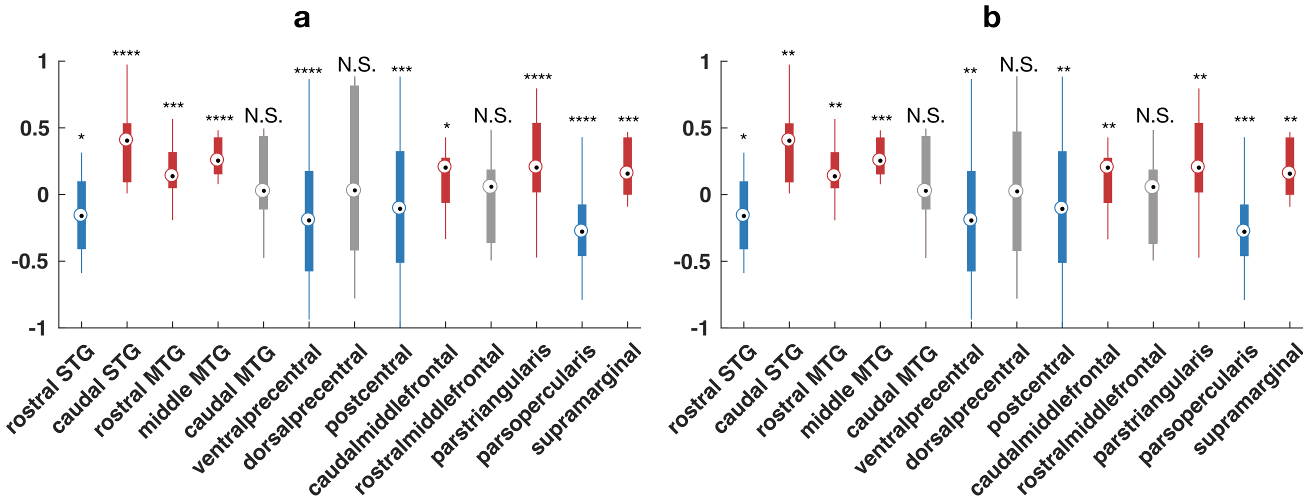

Fig. S9. Statistical comparison of causality for each region controlling for an effect of the subject. The contrast is obtained by taking the difference of the anti-causal and causal contribution maps (red means higher anti-causal contribution, while blue means higher causal contribution). Bar plots of causality for each anatomical region are shown with statistical tests annotated using a Wilcoxon sign rank test (a) as well as a one-way ANOVA with subject as a random effect (b) (*: P-value$<$0.05, **: P-value$<$0.01,***: P-value$<$0.001,****: P- value$<$0.0001). The close match of the two statistical approaches rules out the possibility that the Wilcoxon approach is driven by specific subjects. **Related to Figure 3**

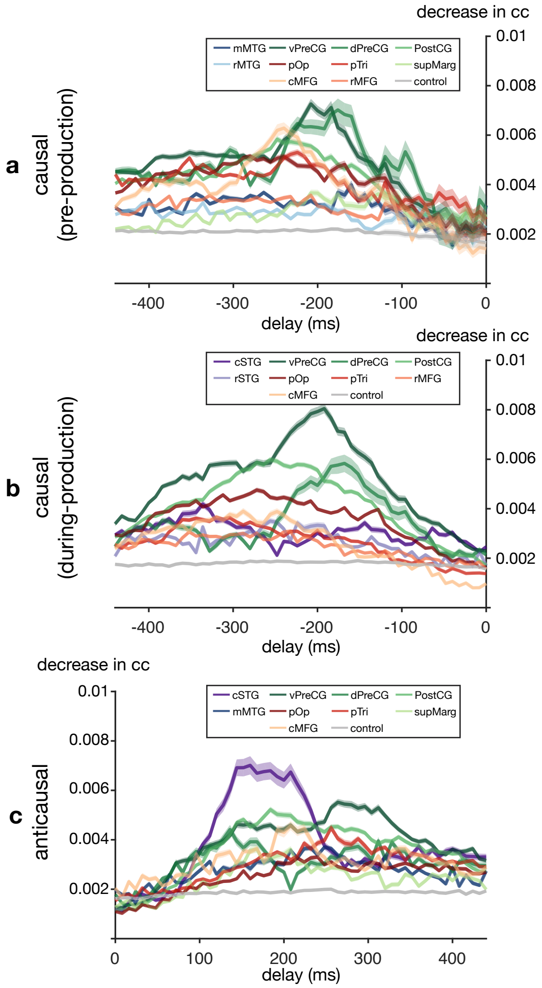

Fig. S10. The non-smoothed temporal receptive field across anatomical region. (a) and (b) are the feedforward temporal receptive fields derived from the causal model by evaluating the contribution of past (negative delays) neural signals during a period before production onset (a) and after onset (b). (c) represents the feedback temporal receptive fields derived from the anticausal models that evaluate the contribution of future (positive delays) neural signals during feedback after articulation. **Related to: Figure 5**

.

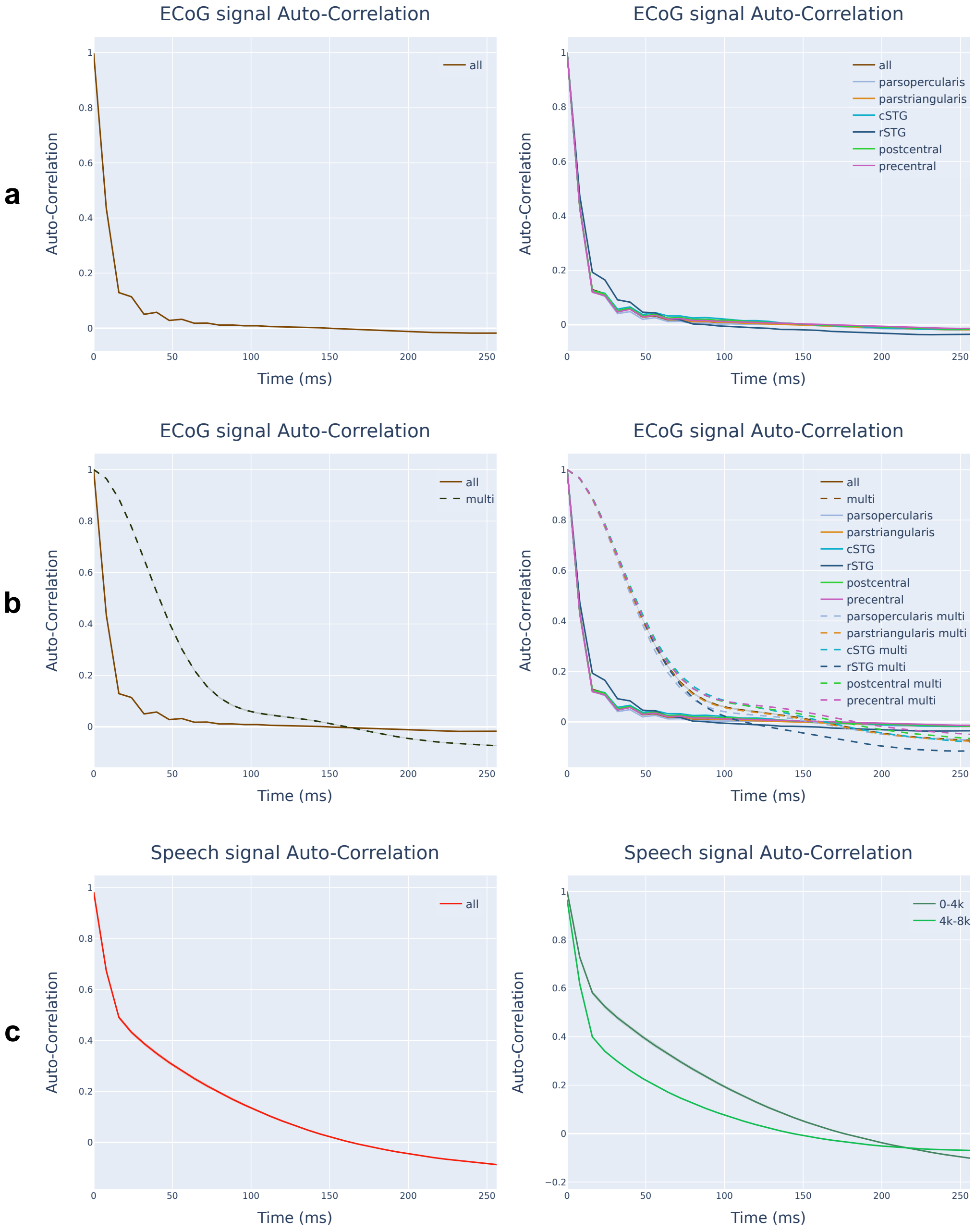

Fig. S11. The auto-correlation of the ECoG signal and Speech spectra signal. (a) The left subfigure shows the auto-correlation of the ECoG signals across all electrode channels, trials, and five subjects. The right subfigure shows the auto-correlation of the ECoG signals across electrodes within specific regions across trials and subjects. The auto-correlation drops to near zero rapidly before 50 ms. (b) Shows the comparison of the auto-correlation of the ECoG signal processed by two methods. The first is a one frequency domain filter (70-150 Hz) used in our study and the second is commonly used by other groups and averages multiple sub-bands within high gamma and is described in (2–4). We show that our choice of filtering reduces the effect of temporal auto-correlation (solid line). (c) Shows the auto-correlation of the speech spectra signal, which drops to near zero before 150 ms. We choose a correlation cutoff of 0.05 to quantify near-zero correlation which provided temporal estimates as follows: 48 ms for our ECoG signal, 112 ms for the multiband approach, 136 ms for the Speech signal.

Anatomical region	p	z
rSTG	0.0332	-2.9628
cSTG	1.607E-15	9.6234
rMTG	2.5108E-04	4.9359
mMTG	1.5257E-13	9.0185
cMTG	0.2269	1.5656
ventralprecentral	4.9511E-8	-7.1409
dorsalprecentral	0.4349	0.6525
postcentral	6.419E-04	-4.9612
cMFG	0.0248	3.1417
rMFG	0.1988	1.7202
parstriangularis	2.6715E-06	6.3518
parsopercularis	8.0693E-15	-9.6185
supramarginal	1.1144E-04	5.3919

Table S1. Statistics of data in Figure 3f. The P-value and Z-value are reported for the Wilcoxon sign rank test between feedback and feedforward contributions across all electrodes and test trials within each anatomical region. The Z-value represents the rank-based test statistic, with positive values reflecting anti-causal contributions and negative values reflecting causal contributions. Related to Figure 3

	Causal pre		Causal during		Anti-causal	
**Anatomical region**	**p-value**	**z-value**	**p-value**	**z-value**	**p-value**	**z-value**
cSTG	0.7226	0.075	5.6745E-10	8.4078	1.2168E-22	10.9874
rSTG	0.4942	-0.0510	1.2703E-07	6.8284	0.1557	1.9149
mMTG	5.3721E-06	6.2146	0.3689	0.2741	1.8216E-28	12.1658
cMTG	0.1671	1.0126	0.231	-0.501	0.4781	-0.3012
rMTG	5.1253E-19	10.1547	0.1293	2.1082	1.4923E-10	8.2051
ventralprecentral	1.7845E-58	16.2047	3.0286E-77	17.5451	2.2394E-60	17.1839
dorsalprecentral	2.9083E-12	8.0932	8.9452E-04	4.4590	1.4512E-09	7.9235
postcentral	3.67853E-91	21.4986	9.34051E-104	22.1393	6.9834E-34	14.0134
supramarginal	2.2905E-06	6.7810	0.5924	-0.2945	1.8542E-07	6.9384
parsopercularis	3.9368E-76	19.0572	3.843E-72	18.5329	3.083E-04	5.3823
parstriangularis	7.2744E-77	19.5782	5.8573E-31	13.9374	2.0273E-37	14.4676
rMFG	2.3846E-27	12.2940	2.0371E-07	7.8460	0.3643	0.3823
cMFG	4.0274E-26	11.0042	2.83632E-07	6.9027	9.02834E-19	9.1881

Table S2. Statistics of data in Figures 4 and 5. Per anatomical region, P-value and Z-value are reported for the Wilcoxon sign rank test between each region's contribution and the shuffled model's contribution (control curves). The Z-value represents the rank-based test statistic, with positive values reflecting larger real contributions compared with shuffled contributions. This is shown for the causal model (pre-production period), causal model (during production period), and anti-causal model, respectively. Curves of each electrode and test trial are considered one sample and averaged across time to perform the Wilcoxon sign rank test. The red marked regions in the table are highlighted to show no significance (P-value$>$0.05) and are omitted when plotting the curves in Figure 5 as described (see Method sections Revealing delay-dependent decoding contributions on cortex, Visualizing spatial-temporal contribution receptive fields). Related to Figures 4,5

	Causal vs. Anti-causal		Causal during vs pre	
**Anatomical region**	**P-value**	**Z-value**	**P-value**	**Z-value**
cSTG	2.6789E-17	9.6711	4.718E-04	3.696
rSTG	0.0343	-2.9457	6.2075E-04	4.7427
mMTG	3.2252E-13	9.0928	4.5863E-04	-4.0475
cMTG	0.3930	1.0021	0.2718	-1.1957
rMTG	1.8511E-04	5.1625	1.0173E-10	-8.9283
ventralprecentral	2.8012E-15	-10.0562	8.2757E-05	5.0475
dorsalprecentral	0.6492	0.2967	5.5615E-04	-3.4394
postcentral	3.0581E-08	-6.1286	0.3037	1.7462
supramarginal	1.9928E-07	6.0301	4.8257E-06	-6.0274
parsopercularis	8.6228E-18	-10.0274	0.5922	0.1582
parstriangularis	0.0162	3.9003	3.2532E-32	-12.4583
rMFG	0.0021	-4.9475	2.5714E-04	-5.0131
cMFG	0.0045	3.9862	3.0747E-09	-7.0652

Table S3. Statistics of data in Figures 4 and 5. Per anatomical region, P-value and Z-value are reported for Wilcoxon sign rank test between the causal (during production period) model and the anti-causal model (The positive/negative Z-values represent the direction of the contribution where positive values denote anti-causal greater than causal), as well as the causal model between during- and pre- epochs (The positive/negative Z-values represent the direction of the contribution where positive values denote during production greater than pre-production). Curves of each individual electrode and test trial are considered as one sample and are averaged across the time epoch to perform the Wilcoxon sign rank test. The red-marked regions in the table are highlighted to denote no significance (P-value$>$0.05). Related to Figures 4,5

Anatomical region	Causal (pre)	Causal (during)	Anti-causal
cSTG	-	-352	168
rSTG	-	-256	-
mMTG	-176	-	240
cMTG	-	-	-
rMTG	-192	-	312
ventralprecentral	-196	-208	280
dorsalprecentral	-192	-184	144
postcentral	-248	-256	192
supramarginal	-120	-	184
parsopercularis	-248	-280	232
parstriangularis	-240	-336	264
rMFG	-248	-304	-
cMFG	-248	-304	208

Table S4. Peak time of each anatomical region curves in Figure 5 a,b,c. Each column reports the peak time of the temporal receptive field curves for the causal model (pre-production), causal model (during production), and anti-causal model, respectively. Each region's peak is calculated based on the averaged curve shown in Figure 5 (averaged across trials and electrodes within the region and smoothed, see Figure S10 for unsmoothed data). Related to Figure 5

Anatomical region	p	F
rSTG	3.77E-02	25.06
cSTG	7.88E-05	125.49
rMTG	1.27E-04	783.37
mMTG	4.10E-05	2436.71
cMTG	1.11E-01	7.514
ventralprecentral	2.23E-04	447.88
dorsalprecentral	2.09E-01	3.35
postcentral	2.56E-03	388.99
cMFG	7.41E-03	133.37
rMFG	1.54E-01	16.35
parstriangularis	4.66E-03	213.32
parsopercularis	1.71E-05	5865.19
supramarginal	1.75E-03	571.71

Table S5. Statistics of data in Figure S9. The P-value and F-value are reported for an ANOVA test between feedback and feedforward contributions across all electrodes and test trials within each anatomical region. Related to Figures 3, S9

Movie S1. Decoded samples (original speech followed by decoded speech).

Movie S2. Decoded samples (decoded speech followed by original speech).

References

1. P Roussel, et al., Observation and assessment of acoustic contamination of electrophysiological brain signals during speech production and sound perception. *J. Neural Eng.* **17**, 056028 (2020).
2. Y Oganian, EF Chang, A speech envelope landmark for syllable encoding in human superior temporal gyrus. *Sci. advances* **5**, eaay6279 (2019).
3. K Rupp, et al., Neural responses in human superior temporal cortex support coding of voice representations. *PLoS Biol.* **20**, e3001675 (2022).
4. S Duraivel, et al., High-resolution neural recordings improve the accuracy of speech decoding. *bioRxiv* (2022).
